# Thermoresponsive Hydrogel‐Enabled Thermostatic Photothermal Therapy for Enhanced Healing of Bacteria‐Infected Wounds

**DOI:** 10.1002/advs.202206865

**Published:** 2023-02-12

**Authors:** Hao Fu, Ke Xue, Yongxin Zhang, Minghui Xiao, Kaiyu Wu, Linqi Shi, Chunlei Zhu

**Affiliations:** ^1^ Key Laboratory of Functional Polymer Materials of Ministry of Education State Key Laboratory of Medicinal Chemical Biology Frontiers Science Center for New Organic Matter College of Chemistry Nankai University Tianjin 300071 China

**Keywords:** bacterial infections, phase transition, photothermal materials, thermoresponsive hydrogels, thermostatic photothermal therapy

## Abstract

Photothermal therapy (PTT) has emerged as an attractive technique for the treatment of bacterial infections. However, the uncontrolled heat generation in conventional PTT inevitably causes thermal damages to healthy tissues and/or organs. It is thus essential to develop a smart and universal strategy to regulate the photothermal equilibrium temperature to a preset safe threshold. Herein, a thermoresponsive hydrogel‐enabled thermostatic PTT system for enhanced healing of bacteria‐infected wounds is reported. In this system, the near‐infrared (NIR)‐triggered heat generation by photothermal nanomaterials is spontaneously transferred to a thermoresponsive hydrogel with a lower critical solution temperature (LCST), leading to its rapid phase transition by forming considerable light‐scattering centers to block NIR penetration. Such a dynamic and reversible process automatically regulates the photothermal equilibrium temperature to the phase‐transition point of the LCST‐type hydrogel. In contrast to temperature‐uncontrolled conventional PTT with severe thermal damages, the thermoresponsive hydrogel‐enabled thermostatic PTT provides effective protection on healthy tissues and/or organs, which remarkably accelerates wound healing by efficient bacterial eradication. This study establishes a smart, simple and universal PTT platform, holding great promise in the safe and efficient treatment of bacterial skin infections.

## Graphical abstract

A thermoresponsive hydrogel‐enabled thermostatic photothermal therapy system is reported for enhanced healing of bacteria‐infected wounds. Taking advantage of the reversible phase transition properties of the hydrogel, the system photothermal equilibrium temperature can be automatically regulated to the phase‐transition point of the hydrogel, which is employed for smart photothermal therapy with negligible thermal damages to healthy tissues and/or organs.

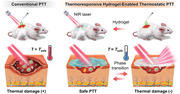

## 1 Introduction

Being one of the major threats to human health, bacterial infections have exerted a huge burden on individuals, the society and the economy.^[^ ^1^, ^2^, ^3^ ^]^ Although the discovery of antibiotics provides human a powerful weapon to fight against bacteria, the misuse and overuse of antibiotics lead to the rapid evolution of drug‐resistant bacteria.^[^ ^4^, ^5^, ^6^, ^7^ ^]^ It is thus of great significance to develop antibiotic‐independent approaches for efficient antibacterial therapy.^[^ ^8^, ^9^, ^10^, ^11^ ^]^ Among various antibiotic‐independent strategies, photothermal therapy (PTT) have emerged as an attractive technique for the treatment of bacterial infections due to its unique advantages, such as non‐invasiveness, high selectivity, broad‐spectrum antibacterial activities and easy eradication of drug‐resistant bacteria (Table S1, Supporting Information).^[^ ^12^, ^13^, ^14^, ^15^ ^]^ PTT primarily utilizes the largely generated heat by photothermal materials under near‐infrared (NIR) light to inactivate bacteria by damaging bacterial membranes and/or denaturing bacterial proteins.^[^ ^16^, ^17^, ^18^ ^]^ Although tremendous achievements have been made in the past decades, conventional PTT lacks an inner self‐regulation mechanism for precise temperature control, which inevitably causes thermal damages to healthy tissues and/or organs.^[^ ^19^, ^20^, ^21^, ^22^ ^]^ To alleviate the adverse effects resulting from uncontrolled heat generation, mild PTT at a temperature of <50 °C has been increasingly adopted by adjusting various experimental parameters, such as the concentration of photothermal materials, the laser power density and the irradiation time.^[^ ^23^, ^24^, ^25^, ^26^ ^]^ However, such a procedure is cumbersome, and the repeatability on the control of photothermal temperature is poor; moreover, an oversight on the adjustment of external experimental parameters can bring instantaneous overheating to initiate irreversible thermal damages. As such, it is highly preferred to design a universal PTT platform that can automatically shut off the photothermal conversion process when the temperature reaches a preset value for safe and efficient treatment of bacterial infections.

Taking advantage of the reversible phase‐transition properties, thermoresponsive polymers with a lower critical solution temperature (LCST) have been widely used as a class of smart materials for a plethora of biomedical applications, such as controlled drug delivery, tissue engineering and biosensing.^[^ ^27^, ^28^, ^29^ ^]^ In general, LCST‐type thermoresponsive polymers are miscible with an appropriate solvent at low temperatures and undergo coil‐to‐globule transition (i.e., phase separation) at temperatures higher than the phase‐transition point (i.e., the cloud point temperature at a given value of transmittance, *T*_cp_).^[^ ^30^, ^31^, ^32^, ^33^ ^]^ Among various LCST‐type thermoresponsive polymers, poly(*N*‐isopropylacrylamide) (PNIPAM) is the most commonly used one due to its water solubility and the proximity of *T*_cp_ to human body temperature.^[^ ^34^, ^35^, ^36^ ^]^ During phase transition, PNIPAM hydrogels exhibit a series of changes with respect to their physicochemical properties, including hydrogen bonding, hydrophobicity and transmittance/reflectivity, in which the variation in light transmittance can serve as a reversible switch to limit the photothermal equilibrium temperature to the *T*_cp_ of PNIPAM hydrogels.^[^ ^37^, ^38^ ^]^ Specifically, when the system temperature is lower than *T*_cp_, the PNIPAM hydrogel appears as a highly transparent semi‐solid due to the formation of hydrogen bonds with water molecules. In contrast, when the system temperature is higher than *T*_cp_, the PNIPAM hydrogel turns into an opaque white block due to dehydration.^[^ ^39^, ^40^ ^]^ During this process, a large number of light‐scattering centers are formed, leading to efficient blocking on NIR penetration.^[^ ^41^, ^42^, ^43^ ^]^ Such an attractive feature can be employed for the design of a smart PTT system with a built‐in temperature‐control mechanism.

**Scheme 1:**
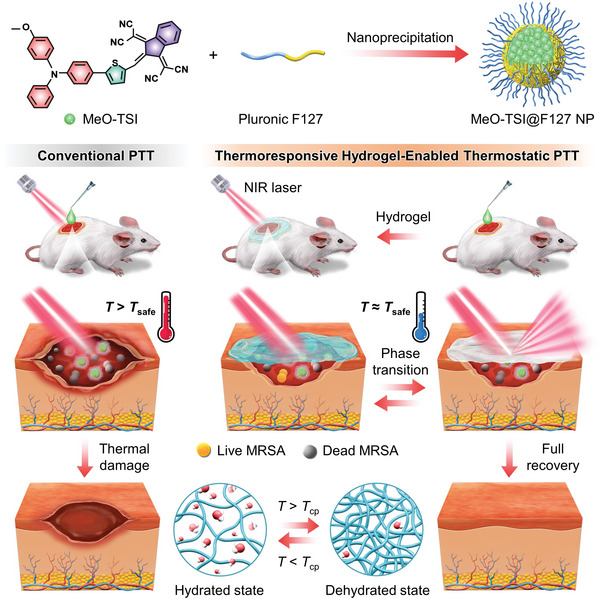
Schematic illustration showing the thermoresponsive hydrogel‐enabled thermostatic PTT with negligible thermal damages for enhanced healing of bacteria‐infected wounds.

In this work, we reported a thermoresponsive hydrogel‐enabled thermostatic PTT system with negligible thermal damages to healthy tissues and/or organs for enhanced healing of bacteria‐infected wounds (**Scheme** **1**). First of all, an NIR‐absorbing aggregation‐induced emission (AIE) photothermal agent was designed and synthesized, which was formulated into colloidally stable nanoparticles (denoted as MeO‐TSI@F127 NPs) using a nanoprecipitation method. To tune the *T*_cp_ of PNIPAM hydrogels into the therapeutic window (i.e., 45–50 °C), a hydrophilic monomer, acrylamide (AM), was introduced to copolymerize with NIPAM to give P(NIPAM‐AM) hydrogels. Next, we validated the mechanism of P(NIPAM‐AM) hydrogel‐enabled PTT for smart and precise temperature control. Upon NIR irradiation, the largely generated heat by MeO‐TSI@F127 NPs was transferred to the P(NIPAM‐AM) hydrogel, leading to its rapid phase transition to block NIR penetration. Such a dynamic and reversible process automatically adjusted the photothermal equilibrium temperature to the *T*_cp_ of P(NIPAM‐AM) hydrogels. In vivo antibacterial studies demonstrated that in contrast to conventional PTT with severe thermal damages, no evident adverse effects to healthy tissues and/or organs were found in P(NIPAM‐AM) hydrogel‐enabled thermostatic PTT, which significantly accelerated the recovery of the infected wounds by efficient bacterial eradication. To the best of our knowledge, this study is the first report that utilizes the phase‐transition properties of LCST‐type hydrogels to regulate the heat‐generation behaviors of photothermal materials for the safe treatment of bacterial skin infections.

## 2 Results and Discussion

**Figure 1:**
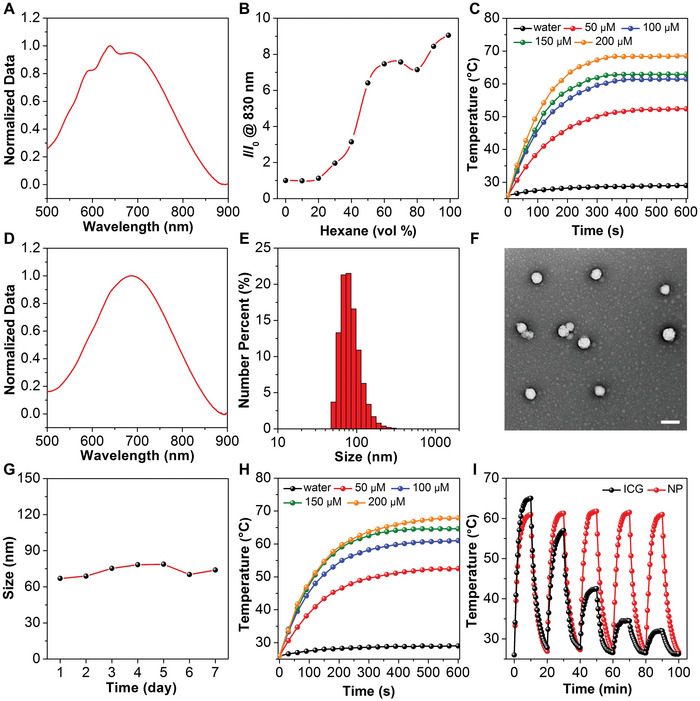
Characterizations on the physiochemical properties of MeO‐TSI. A) Normalized absorption spectrum of MeO‐TSI nanoaggregates. B) Plot of relative emission intensity (*I*/*I*_0_@830 nm) as a function of hexane fraction in CHCl_3_/hexane mixtures. C) Photothermal curves of MeO‐TSI nanoaggregates with different concentrations irradiated by an 808 nm laser at a power density of 1.2 W cm^−2^. D) Normalized absorption spectrum of MeO‐TSI@F127 NPs. E) Size distribution of MeO‐TSI@F127 NPs measured by DLS. F) TEM image of MeO‐TSI@F127 NPs. Scale bar: 100 nm. G) Size stability of MeO‐TSI@F127 NPs. H) Photothermal curves of MeO‐TSI@F127 NPs with different concentrations irradiated by an 808 nm laser at a power density of 1.2 W cm^−2^. I) Photothermal stability of MeO‐TSI@F127 NPs (100 µm) and ICG (100 µm) after five cycles of repeated irradiation by an 808 nm laser at a power density of 1.2 W cm^−2^.

To overcome the photobleaching issue encountered by traditional photothermal agents, an AIE photothermal agent (denoted as MeO‐TSI) was designed and synthesized, in which a typical donor–*π*–acceptor (D–*π*–A) structure was constructed to red‐shift the absorption and emission wavelengths into the NIR region. The synthetic route to MeO‐TSI is shown in Figure S1, Supporting Information. Specifically, compound 1 and 5‐formyl‐2‐thiopheneboronic acid were reacted under Suzuki coupling conditions to give compound 2. Next, 1,3‐bis(dicyanomethylidene)indane was attached to compound 2 via Knoevenagel condensation to obtain MeO‐TSI. The chemical structures of all compounds were verified by nuclear magnetic resonance spectrometry and high‐resolution mass spectrometry (Figures S2–S7, Supporting Information). We first performed basic physiochemical characterizations on MeO‐TSI. As shown in **Figure** **1**A, MeO‐TSI had a major absorption at ca. 650 nm with the peak tail extending to the NIR region, which is preferred as NIR light provides deeper penetration into biological tissues to maximize therapeutic efficacy. Due to the introduction of multiple molecular rotors, MeO‐TSI showed a typical AIE property.^[^ ^44^, ^45^, ^46^, ^47^, ^48^ ^]^ With the increase of hexane in CHCl_3_, there was an obvious enhancement in the emission intensity of MeO‐TSI at 830 nm, with the enhancement ratio (i.e., *I*/*I*_0_@830 nm) reaching ca. 9 when the fraction of hexane was increased to 99% (Figure 1B and Figure S8, Supporting Information). To examine the photothermal behaviors of MeO‐TSI in aqueous solution, the dimethyl sulfoxide (DMSO) solution of MeO‐TSI was added into water to give MeO‐TSI nanoaggregates (Figure S9, Supporting Information). As shown in Figure 1C and Figure S10, Supporting Information, MeO‐TSI nanoaggregates exhibited a concentration‐ and laser power‐dependent heat generation under NIR irradiation, and the measured photothermal equilibrium temperature ranged from ca. 35 to 71 °C. By referring to a previous publication, the photothermal conversion efficiency of MeO‐TSI nanoaggregates was determined to be 40.3% (Figure S11, Supporting Information). To favor biomedical applications, a biocompatible polymer (i.e., Pluronic F127) was employed as the amphiphile to formulate the hydrophobic MeO‐TSI into colloidally stable nanoparticles (denoted as MeO‐TSI@F127 NPs) via nanoprecipitation. In contrast to MeO‐TSI nanoaggregates, MeO‐TSI@F127 NPs exhibited a smoother and slightly red‐shifted absorption spectrum, with the absorption wavelength ranging from 500–900 nm (Figure 1D). Dynamic light scattering (DLS) result showed that the hydrodynamic diameter of MeO‐TSI@F127 NPs was primarily distributed between 50–200 nm, with the majority locating at 60–100 nm (Figure 1E). Transmission electron microscopy (TEM) demonstrated that MeO‐TSI@F127 NPs had a spherical morphology with an average diameter of ca. 70 nm, which was basically consistent with the DLS data (Figure 1F). During 7‐day storage, no evident change in the hydrodynamic diameters of MeO‐TSI@F127 NPs was found, suggesting the excellent particle stability (Figure 1G). Next, the photothermal properties of MeO‐TSI@F127 NPs were evaluated using the identical conditions as tested for MeO‐TSI nanoaggregates. Similarly, MeO‐TSI@F127 NPs showed concentration‐ and laser power‐dependent photothermal behaviors upon NIR irradiation, with the equilibrium temperatures ranging from ca. 36 to 72 °C (Figure 1H and Figure S12, Supporting Information). Further calculation indicated that MeO‐TSI@F127 NPs had a slightly higher photothermal conversion efficiency (44.2%) than MeO‐TSI nanoaggregates (Figure S13, Supporting Information), which was probably attributed to the improved utilization of the NIR laser due to the slightly red‐shifted absorption. Furthermore, we compared the photothermal stability of MeO‐TSI@F127 NPs with a classical NIR dye indocyanine green (ICG). As shown in Figure 1I, after five cycles of repeated NIR irradiation, the photothermal performance of MeO‐TSI@F127 NPs remained unchanged, whereas the net temperature difference for ICG was decreased from ca. 40 to 7 °C due to photobleaching, suggesting the superiority of AIE photothermal materials over traditional ones.

**Figure 2:**
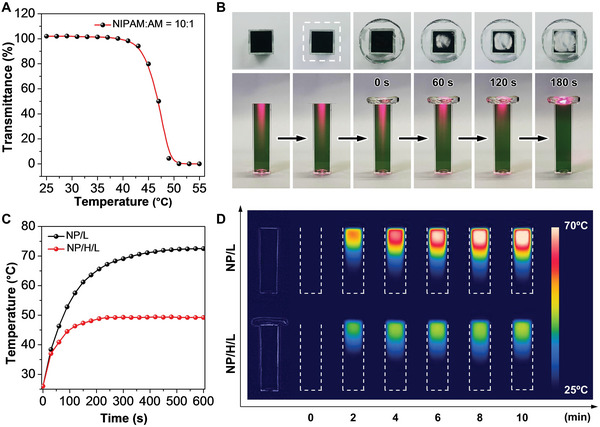
Smart control on the photothermal behaviors of MeO‐TSI@F127 NPs in the presence of a P(NIPAM‐AM) hydrogel (NIPAM:AM = 10:1). A) Transmittance of the hydrogel at 808 nm as a function of temperature. B) Photographs showing the changes in the appearance of the hydrogel and the penetration of an 808 nm laser into the dispersion. C) Photothermal curves of MeO‐TSI@F127 NPs (200 *µ* m) in the absence (NP/L) and presence (NP/H/L) of the hydrogel upon NIR irradiation (1.5 W cm^−2^). D) Thermal images of MeO‐TSI@F127 NPs (200 *µ* m) in the absence (NP/L) and presence (NP/H/L) of the hydrogel upon NIR irradiation (1.5 W cm^−2^).

To regulate the photothermal behaviors of MeO‐TSI@F127 NPs, three LCST‐type thermoresponsive hydrogels were fabricated, in which NIPAM and AM were used as the monomers, and *N*,*N*′‐methylene bisacrylamide (MBA) served as the crosslinker. We first measured the transmittance of the resulting P(NIPAM‐AM) hydrogels at 808 nm as a function of temperature to determine the *T*_cp_ at 5% transmittance. As shown in **Figure** **2**A and Figure S14, Supporting Information, as the mass ratio of NIPAM and AM increased from 10:1, 30:1, to 50:1, the measured *T*_cp_ decreased from 48.9, 44.6, to 40.4 °C, which was attributed to the reduced hydrogen bonding with water. Taking the P(NIPAM‐AM) hydrogel (NIPAM:AM = 10:1) as an example, we collected the intuitive photographs to show the reversible phase‐transition process. As shown in Figure S15, Supporting Information, when the system was heated to a temperature that was higher than *T*_cp_, the transparent hydrogel became whitened and dehydrated. Upon natural cooling, the appearance of the hydrogel was gradually recovered to the original transparent state by absorbing the extruded water. Even when the hydrogel was completely cooled down to room temperature, there was still left a small amount of water. Such a feature was particularly desired for wound treatment as the extruded water could serve as a liquid lubricant to avoid secondary damages on the wound during hydrogel removal. Next, we assessed the capability of these P(NIPAM‐AM) hydrogels on regulating the transmittance of NIR light during phase transition. In our experiment, a coverslip was placed on the top surface of a cuvette filled with MeO‐TSI@F127 NPs to make them physically contact with each other. After capping with a P(NIPAM‐AM) hydrogel, an 808 nm NIR laser was irradiated at a direction perpendicular to the coverslip. In contrast to the situation without any occluded objects, the presence of a coverslip and a transparent hydrogel did not affect NIR penetration into the dispersion of MeO‐TSI@F127 NPs (Figure 2B). In this case, the photothermal conversion of MeO‐TSI@F127 NPs remarkably elevated the system temperature, giving rise to the phase transition of the P(NIPAM‐AM) hydrogel to make it appear as an opaque white block; during this process, the penetration of the NIR light was gradually cut off. When the temperature reached *T*_cp_, the area on the hydrogel that corresponded to the laser spot became completely white, and the laser beam scarcely passed through the hydrogel, which automatically regulated the photothermal equilibrium temperature to the *T*_cp_ of P(NIPAM‐AM) hydrogels. Furthermore, we validated the capability of P(NIPAM‐AM) hydrogels on controlling the photothermal behaviors of MeO‐TSI@F127 NPs with varying concentrations. In the absence of P(NIPAM‐AM) hydrogels, the highest photothermal temperatures spanned from 49.5 to 72.5 °C when the concentration of MeO‐TSI@F127 NPs and the laser power density varied from 100–200 µm and 0.6–1.5 W cm^−2^, respectively (Figure 2C and Figure S16, Supporting Information). In the presence of the P(NIPAM‐AM) hydrogel (NIPAM:AM = 10:1), however, the upper limit of the equilibrium temperature was found to be ca. 49 °C, which was close to the *T*_cp_ of the hydrogel. Such a comparison could also be visualized by the time‐dependent thermal images, suggesting the temperature‐control capability of the P(NIPAM‐AM) hydrogel (Figure 2D). To verify the universality of this strategy, the other two P(NIPAM‐AM) hydrogels (NIPAM:AM = 30:1 and 50:1) were also tested, in which the highest photothermal equilibrium temperature of MeO‐TSI@F127 NPs was set at 20 °C higher than the *T*_cp_ of the hydrogels. As shown in Figures S17 and S18, Supporting Information, the equilibrium temperatures for both systems were all close to the *T*_cp_ of the corresponding hydrogels, indicating the feasibility of using LCST‐type hydrogels for smart and precise temperature control.

**Figure 3:**
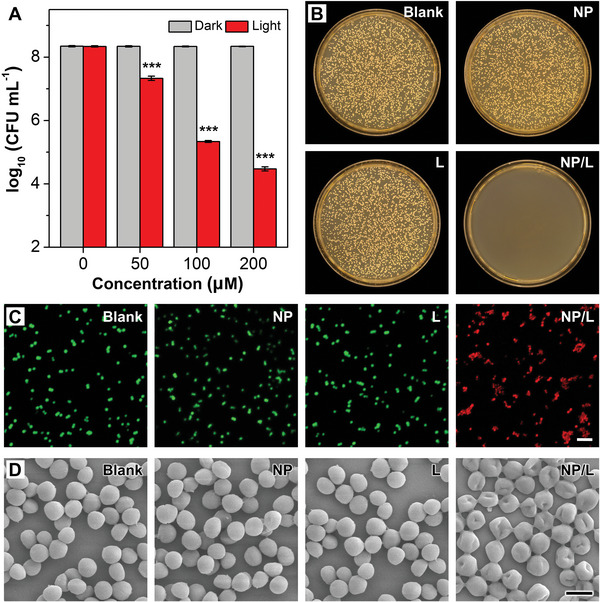
In vitro antibacterial behaviors of MeO‐TSI@F127 NPs toward MRSA upon NIR irradiation. A) Statistical analysis of bacterial viability in the presence of varying concentrations of MeO‐TSI@F127 NPs without and with NIR irradiation (*n* = 3). ***p < 0.001 when compared with the blank group. B) Photographs of bacterial colonies formed on agar plates for different groups. [MeO‐TSI@F127 NPs] = 100 µm. C) CLSM images of live (green) and dead (red) bacteria in different groups. [MeO‐TSI@F127 NPs] = 100 µm. Scale bar: 5 µm. D) SEM images of bacteria in different groups. [MeO‐TSI@F127 NPs] = 100 µm. Scale bar: 1 µm. “Blank”, “NP”, “L” and “NP/L” represent the control group treated with PBS, the group treated with MeO‐TSI@F127 NPs, the NIR‐irradiated “Blank” group and the NIR‐irradiated “NP” group, respectively.

Next, we studied the in vitro temperature‐dependent antibacterial behaviors of MeO‐TSI@F127 NPs toward the clinically relevant Gram‐positive methicillin‐resistant *Staphylococcus aureus* (MRSA) by varying the concentration of MeO‐TSI@F127 NPs from 0 to 200 µm in the absence and presence of NIR irradiation. As shown in **Figure** **3**A,B and Figure S19, Supporting Information, no obvious antibacterial activities were found for all groups without NIR irradiation. In contrast, upon NIR irradiation, there was a temperature‐dependent antibacterial effect, with the bactericidal efficiencies of ca. 90%, 99.9% and 99.99% when the concentration of MeO‐TSI@F127 NPs was set at 50, 100 and 200 µm, respectively; under such conditions, the corresponding photothermal equilibrium temperature was ca. 43, 49 and 56 °C, respectively (Figure S12B, Supporting Information). Considering the possible bacterial loss during hydrogel removal and incomplete contact with the bottom bacterial suspension, the hydrogel was not directly applied in the in vitro experiments. Nevertheless, to simulate the antibacterial behaviors of MeO‐TSI@F127 NPs in the presence of the hydrogel with a *T*_cp_ of ca. 49 °C, the experimental parameter was carefully adjusted to make the equilibrium temperature locate at ca. 49 °C, in which the concentration of MeO‐TSI@F127 NPs was set at 100 µm. Live/dead fluorescent staining was then performed to visualize the antibacterial effects of MeO‐TSI@F127 NPs at 100 µm. As shown in Figure 3C, all bacteria exhibited green fluorescence for the groups treated with phosphate‐buffered saline (PBS), MeO‐TSI@F127 NPs and NIR irradiation alone. In contrast, NIR irradiation transformed almost all green‐emissive bacteria to red‐emissive ones for the group treated with MeO‐TSI@F127 NPs, which was in accordance with the results of the plate‐counting assay. Furthermore, scanning electron microscopy (SEM) was employed to characterize the morphological changes of bacteria under different treatments. As shown in Figure 3D, for the groups treated with PBS, MeO‐TSI@F127 NPs and NIR irradiation alone, almost all bacteria kept a relatively intact and smooth cell body. In contrast, pronounced collapse and fusion in bacterial membranes were observed when MeO‐TSI@F127 NPs and NIR irradiation were simultaneously imposed, which was attributed to the hyperthermia‐mediated disruption of bacterial structures. In addition, we also evaluated the dark toxicity of MeO‐TSI@F127 NPs to a normal fibroblast cell line NIH‐3T3 (Figure S20, Supporting Information), and no significant inhibitory effect on the cell viability was observed, suggesting the biocompatibility of MeO‐TSI@F127 NPs to normal mammalian cells. In addition, we also evaluated the viability of NIH‐3T3 cells under different photothermal equilibrium temperatures for different periods of time. As shown in Figure S21, Supporting Information, the cells maintained a high viability (>80%) when treated with a photothermal equilibrium temperature of 49 °C for 5 min. When the irradiation time was extended to 10 min, the cell viability was reduced to ca. 40%. In contrast, when the treatment conditions were changed to a photothermal equilibrium temperature of 61 °C for 5 and 10 min, the corresponding cell viabilities were only ca. 10%. To minimize the adverse impact of high temperature on cell viability, the NIR irradiation time was limited to 5 min for in vivo experiments.

**Figure 4:**
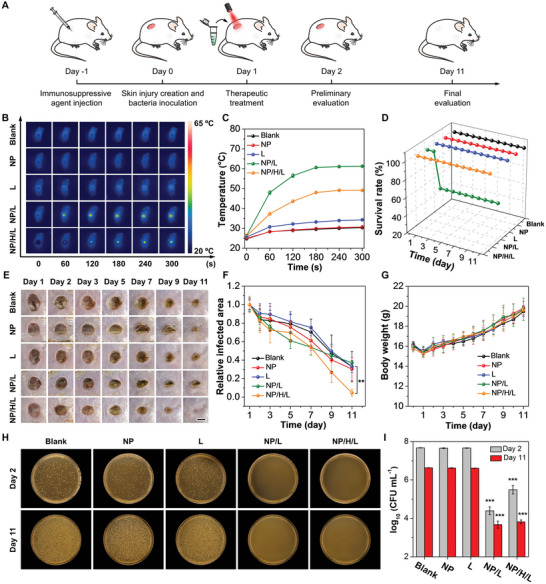
In vivo antibacterial behaviors of MeO‐TSI@F127 NPs (150 µm) with P(NIPAM‐AM) hydrogel‐enabled temperature control upon NIR irradiation. A) Schematic illustration showing the timeline of the evaluation period. B) Thermal images of mice under different treatments. C) Temperature profiles extracted from the corresponding thermal images (*n* = 5). D) Survival rate of mice under different treatments as a function of time (for the “NP/L” group, *n* = 5 before and at day 2, *n* = 3 after day 2; for all the other groups, *n* = 5). E) Photographs of the bacteria‐infected wounds under different treatments. Scale bar: 5 mm. F) Relative infected areas of mice under different treatments (for the “NP/L” group, *n* = 5 before and at day 2, *n* = 3 after day 2; for all the other groups, *n* = 5). G) Body weights of mice under different treatments (for the “NP/L” group, *n* = 5 before and at day 2, *n* = 3 after day 2; for all the other groups, *n* = 5). H) Photographs of bacterial colonies formed on agar plates for different groups after the completion of in vivo antibacterial experiments. The dilution time for the groups at day 2 and 11 was 1 × 10^4^ and 1 × 10^2^, respectively. I) Statistical analysis of the bacterial viability by log_10_ (CFU mL^−1^). “Blank”, “NP”, “L”, “NP/L” and “NP/H/L” represent the control group treated with PBS, the group treated with MeO‐TSI@F127 NPs, the NIR‐irradiated “Blank” group, the NIR‐irradiated “NP” group and the group treated with MeO‐TSI@F127 NPs and the P(NIPAM‐AM) hydrogel (NIPAM:AM = 10:1) under NIR irradiation (*n* = 3). **p < 0.01 and ***p < 0.001 when compared with the “Blank” group.

Motivated by the in vitro antibacterial results, an animal model with bacterial infections was established to evaluate the in vivo therapeutic effects of the smart PTT system, in which the P(NIPAM‐AM) hydrogel (NIPAM:AM = 10:1) with a *T*_cp_ of ca. 49 °C was used to ensure the bactericidal performance while avoiding evident thermal damages to the surrounding healthy tissues and/or organs. Specifically, a round skin wound was created on the back of each mouse, followed by the inoculation of MRSA for bacterial infections. The infected mice were randomly divided into five groups for different treatments (**Figure** **4**A). During NIR irradiation, the temperature was dynamically monitored by an infrared camera. Notably, in view of the shallow wound depth, the concentration of MeO‐TSI@F127 NPs was increased to 150 µm to offset the small volume (50 µL) applied on the infected wounds. As shown in Figure 4B,C, in contrast to the groups without NIR irradiation, there was only a slight temperature increase for the group treated with PBS upon NIR irradiation. However, the temperature in the group treated with MeO‐TSI@F127 NPs and NIR irradiation (denoted as “NP/L”) exhibited a sharp temperature gradient in the wound area, with a value of up to 61 °C after laser irradiation. Interestingly, the application of the P(NIPAM‐AM) hydrogel effectively unified temperature distribution in the wound area, with the temperature equilibrating at ca. 49 °C. The smart temperature control was attributed to the phase transition‐mediated NIR blocking, which was authenticated by the whitened hydrogel during NIR irradiation (Figure S22, Supporting Information). Notably, the presence of the dispersion of MeO‐TSI@F127 NPs facilitated the close adhesion of the hydrogel with the wound due to the surface tension of water. Subsequently, we recorded the changes in the survival rate, infected area and body weight of the mice throughout the evaluation period (11 days). As shown in Figure 4D, the survival rate for the “NP/L” group dropped to 60% on the following day after treatments (i.e., day 2), while the mice in all the other groups survived the entire therapeutic period. We speculated that the drastic decrease in the survival rate resulted from the uncontrolled hyperthermia‐caused thermal damages, which severely affected the biological functions of adjacent tissues and/or major organs.^[^ ^19^ ^]^ In terms of wound healing, although all groups showed reduced infected areas over time, the recovery rate for the “NP/L” group was slightly slower than that of the blank group (Figure 4E,F). Notably, the wound in the “NP/L” group became blackened at day 2 and formed empyrosis‐induced eschars at day 5, which was distinct from regular scabs as seen in the other groups. Surprisingly, the application of the hydrogel to the “NP/L” group (denoted as “NP/H/L”) remarkably promoted the healing of the infected wounds, suggesting the advantage of the hydrogel‐enabled smart PTT for wound recovery. During the therapeutic period, the body weights of all mice showed an increasing trend except for day 1, which was attributed to the creation of injuries and bacterial infections (Figure 4G). To preliminarily evaluate the antibacterial effects, one of the mice in each group was randomly selected and sacrificed at day 2, and half of the infected tissues were collected for bacterial quantification. At the end of the therapeutic period, all surviving mice were sacrificed, and half of the infected tissues were homogenized for bacterial quantification. As shown in Figure 4H,I, in contrast to the preliminary antibacterial results at day 2, there was one order of magnitude decrease in CFU numbers at day 11 for all the groups without PTT. In terms of the “NP/L” group, NIR irradiation gave rise to three orders of magnitude decrease (corresponding to a killing efficiency of 99.9%) at day 2 and 11, respectively. As for the “NP/H/L” group, although there were only two orders of magnitude decrease (corresponding to a killing efficiency of 99%) at day 2, the difference in the antibacterial efficacy between the “NP/L” and “NP/H/L” groups was significantly lessened at day 11, suggesting the accelerated recovery of wound infection.

**Figure 5:**
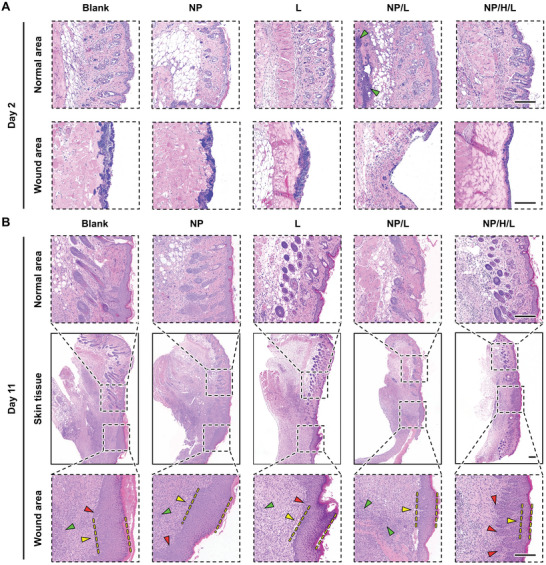
Histological analysis of infected wounds under different treatments stained by H&E. A) Day 2. B) Day 11. Green, yellow and red arrowheads indicate the inflammation, regenerated epidermis and new hair follicles, respectively. Scale bars: 200 µm.

To gain more insight into thermal damages, we performed histological analysis on the infected wounds and their adjacent tissues. Hematoxylin and eosin (H&E) staining at day 2 showed that except for the “NP/L” group, there were noticeable inflammatory responses on the surfaces of the infected wounds, with the “NP/H/L” group exhibiting the lowest degree of inflammation (**Figure** **5**A). In terms of the “NP/L” group, although the inflamed top surface disappeared, the histological structure in the dermis was severely lost due to thermal damages, which was characterized by pronounced karyorrhexis and cytolysis. In addition, an evident inflammation reaction was identified in the adjacent normal tissues, which was attributed to the inevitable thermal diffusion from the irradiation center with a temperature of up to 61 °C. In contrast, no pronounced thermal damages were found for the “NP/H/L” group both in the wound area and in the adjacent normal tissue, suggesting the protection effect of the P(NIPAM‐AM) hydrogel on healthy tissues during PTT. After the completion of the therapeutic period, the entire skin that consisted of the wounds and their adjacent normal tissues were collected and half of them were subjected to H&E staining. As shown in Figure 5B, although there remained some inflammatory cells in all groups, the number in the “NP/H/L” group remarkably decreased, suggesting the role of the hydrogel‐enabled smart PTT in lessening inflammatory responses. In general, thinner epidermal layer indicates faster epidermis regeneration. It is clearly seen that the thickness of the new epidermis in the “NP/H/L” group was much thinner than that of all other groups, which was even comparable to the one in the epidermis of normal skin tissues. In addition, the number of new hair follicles is positively correlated with complete wound recovery. Although there were only a small number of immature hair follicles in the groups treated with PBS, MeO‐TSI@F127 NPs and NIR irradiation alone, no hair follicles were found in the wound area in the “NP/L” group. Surprisingly, there were a large number of relatively mature hair follicles in the wound area for the “NP/H/L” group, suggesting the rapid wound recovery and epidermal reconstruction. Besides, we also performed immunohistochemical staining on CD31 (a marker of endothelial cell for vascular differentiation) and collagen (type I and III, denoted as COL I and COL III) to visualize angiogenesis and collagen regeneration. As shown in Figure S23, Supporting Information, in contrast to all the other groups, a large number of neogenerated vessels were observed in the “NP/H/L” group, indicating the faster skin regeneration. Due to the uncontrolled heat generation, there was no evident angiogenesis in the “NP/L” group. Although the positive expression of COL III was much higher than that of COL I for all groups, the amount of COL III and COL I in the “NP/H/L” group was significantly higher than all other groups. Notably, the lowest expression of both COL III and COL I was found in the “NP/L” group, suggesting the poor wound healing as a result of thermal damages. Furthermore, the major organs (including the heart, liver, spleen, lung, and kidney) of mice with different treatments were subjected to histological analysis. As shown in Figure S24, Supporting Information, no obvious pathological abnormalities were found in the sections of the organs of all mice, indicating the biosafety of all materials used in this study.

## 3 Conclusions

In this study, we developed a thermoresponsive hydrogel‐enabled thermostatic PTT system for enhanced healing of bacteria‐infected wounds. The smart system was composed of LCST‐type hydrogels (i.e., P(NIPAM‐AM) hydrogels) and NIR‐absorbing AIE photothermal nanomaterials (i.e., MeO‐TSI@F127 NPs). Upon NIR irradiation, the largely generated heat by MeO‐TSI@F127 NPs initiated the phase transition of the P(NIPAM‐AM) hydrogels, which led to the formation of a large number of light‐scattering centers to block NIR penetration. Such a dynamic and reversible process automatically regulated the photothermal equilibrium temperature to the phase transition point of the thermoresponsive hydrogel. In vivo antibacterial experiments on mice with bacteria‐infected wounds demonstrated that in contrast to temperature‐uncontrolled conventional PTT with severe thermal damages, the hydrogel‐enabled thermostatic PTT provided effective protection on healthy tissues and/or organs, which remarkably accelerated wound healing by efficient bacterial eradication. This study establishes a universal and versatile PTT platform. On the one hand, the thermoresponsive hydrogel‐enabled thermostatic PTT is applicable to almost all photothermal materials, as long as they can produce sufficient heat to trigger phase transition. On the other hand, the photothermal equilibrium temperatures can be arbitrarily tuned simply by changing the ratio of the copolymerized monomers in P(NIPAM‐AM) hydrogels. The smart photothermal system opens up new avenues for the safe implementation of PTT, holding great promise in the efficient treatment of bacterial skin infections in clinical settings.

## Conflict of Interest

The authors declare no conflict of interest.

## Supporting information

Supporting information

## Data Availability

The data that support the findings of this study are available from the corresponding author upon reasonable request.
