# Supplementary material for: Thermoresponsive Hydrogel‐Enabled Thermostatic Photothermal Therapy for Enhanced Healing of Bacteria‐Infected Wounds
Source: Adv Sci (Weinh). 2023 Feb 12;10(11):2206865. doi: 10.1002/advs.202206865 (PMC10104658; doi:10.1002/advs.202206865)
Supplement: Supplementary file 1 — Supporting information [file ADVS-10-2206865-s001.pdf]

ADVANCED SCIENCE
Open Access

Supporting Information

for *Adv. Sci.*, DOI 10.1002/advs.202206865

Thermoresponsive Hydrogel-Enabled Thermostatic Photothermal Therapy for Enhanced Healing of Bacteria-Infected Wounds

*Hao Fu, Ke Xue, Yongxin Zhang, Minghui Xiao, Kaiyu Wu, Linqi Shi and Chunlei Zhu**

Supporting Information

Thermoresponsive Hydrogel-Enabled Thermostatic Photothermal Therapy for Enhanced Healing of Bacteria-Infected Wounds

*Hao Fu, Ke Xue, Yongxin Zhang, Minghui Xiao, Kaiyu Wu, Linqi Shi, and Chunlei Zhu**

H. Fu, Dr. K. Xue, Y. Zhang, M. Xiao, K. Wu, Prof. L. Shi, Prof. C. Zhu

Key Laboratory of Functional Polymer Materials of Ministry of Education, State Key Laboratory of Medicinal Chemical Biology, Frontiers Science Center for New Organic Matter, College of Chemistry, Nankai University, Tianjin 300071, China

Email: chunlei.zhu@nankai.edu.cn

Experimental Section

Chemicals and materials. Tetrakis(triphenylphosphine)palladium ($Pd(PPh_3)_4$), 5-formyl-2-thiopheneboronic acid, and 1,3-bis(dicyanomethylidene)indane were ordered from Shanghai Adamas Reagent Co., Ltd. Potassium persulfate (KPS), tetrahydrofuran (THF), and dimethyl sulfoxide (DMSO) were obtained from Aladdin. Acetic anhydride (Ac_2O), potassium carbonate (K_2CO_3), sodium sulphate (Na_2SO_4), petroleum ether (PE), ethyl acetate (EA), toluene, ethanol (EtOH), and dichloromethane (DCM) were purchased from Tianjin Bohai Chemical Industry Group Co., Ltd. Acrylamide (AM), *N*-isopropylacrylamide (NIPAM), and *N*,*N*'-methylene bisacrylamide (MBA) were obtained from J&K, Heowns, and Fuchen, respectively. Polyethylene-polypropylene glycol (Pluronic F127) and tetramethylethylenediamine (TEMED) were purchased from Energy chemical. 3-(4,5-Dimethylthiazol-2-yl)-2,5-diphenyltetrazolium bromide (MTT) was purchased from Beyotime Biotechnology. Tryptone soy broth (TSB) was purchased from Solarbio. The phosphate buffered saline (PBS, 10 mM, pH = 7.4) was purchased from Thermal Fisher Scientific. All chemicals were used as received without further purification. The water used in all experiments was obtained by filtering through a set of HEAL FORCE cartridges (Smart-N15VF).

Synthesis of compound 2. Compound **1** was synthesized according to a previously published paper.[1] Compound **1** (2.83 mmol, 1.00 g), 5-formyl-2-thiopheneboronic acid (4.25 mmol, 0.66 g), $Pd(PPh_3)_4$ (0.14 mmol, 164 mg), and K_2CO_3 (22.64 mmol, 3.12 g) were placed in a 100 mL round-bottom flask, which was evacuated under vacuum and purged with dry argon gas three times. After adding THF/H_2O (5:1, v/v, 24 mL), the mixture was heated to 70 °C and stirred for 12 h. Next, the mixture was cooled to room temperature, quenched with H_2O, and then extracted with DCM three times. After solvent evaporation under reduced pressure, the crude product was purified on a silica-gel column (PE:EA = 20:1, v/v) to afford compound **2** in 36% yield. ^1H-NMR (400 MHz, $CDCl_3$): δ 9.82 (s, 1H), 7.67 (d, 1H), 7.46 (t, 2H), 7.28–7.23 (m, 3H), 7.08 (dt, 4H), 7.03 (t, 1H), 6.98 (d, 2H), 6.88–6.83 (m, 2H), 3.79 (s, 3H). ^{13}C-NMR (100 MHz, $CDCl_3$): δ 182.60, 156.89, 154.85, 149.53, 147.13, 141.15, 139.79, 137.83, 129.43, 127.88, 127.24, 125.35, 124.41, 123.41, 122.68, 121.06, 115.04, 77.42, 77.10, 76.78, 55.55. HRMS (ESI, m/z, $C_{24}H_{19}NO_2S$, $[M + H^+]$): calcd., 386.1215; found, 386.1208.

Synthesis of MeO-TSI. Compound **2** (2.60 mmol, 1.00 g) and 1,3-bis(dicyanomethylidene)indane (5.20 mmol, 1.26 g) were dissolved in Ac_2O (50 mL),

followed by stirring at 60 °C for 6 h. Next, the mixture was cooled to room temperature. After solvent evaporation under reduced pressure, the crude product was purified on a silica-gel column (PE:EA = 5:1, v/v) to afford MeO-TSI as a black blue solid in 68% yield. ^{1}H-NMR (400 MHz, $CDCl_3$): δ 8.64 (s, 1H), 8.56 (dd, 2H), 7.76 (dd, 2H), 7.64 (d, 1H), 7.49 (d, 2H), 7.31 (dd, 3H), 7.16–7.09 (m, 5H), 6.97 (d, 2H), 6.89 (d, 2H), 3.82 (s, 3H). ^{13}C-NMR (100 MHz, $CDCl_3$): δ 161.00, 158.46, 157.30, 150.69, 146.51, 139.19, 137.37, 136.16, 134.99, 134.52, 129.60, 128.10, 127.86, 126.06, 125.50, 125.09, 124.28, 124.03, 120.23, 115.16, 113.62, 77.45, 77.13, 76.82, 55.59. HRMS (ESI, m/z, $C_{39}H_{23}N_5OS$, [M]): calcd., 609.1623; found, 609.1632.

Characterizations. The absorption spectra of all samples were measured by a UV–Vis–NIR spectrophotometer (Shimadzu, UV-2600). The morphology of all nanoparticles were characterized by transmission electron microscopy (TEM, FEI, Talos F200X G2). The particle size distribution was measured by dynamic light scattering (DLS, Malvern, Zetasizer Nano ZS90).

Preparation of MeO-TSI nanoaggregates. The solution of MeO-TSI in DMSO (1 mM) was poured into water, followed by vigorous vortexing and sonication (5 min) to give the MeO-TSI nanoaggregates. The corresponding fluorescence spectra were characterization by a fluorometer (Edinburgh, FLS1000).

Photothermal properties of MeO-TSI nanoaggregates. The suspensions of MeO-TSI nanoaggregates with varying concentrations of MeO-TSI (50, 100, 150, and 200 µM) were added into a cuvette with a bottom area of 1 cm^2. Next, the systems with different concentrations of MeO-TSI nanoaggregates were irradiated under an 808 nm laser at different power densities (0.3, 0.6, 0.9, 1.2, and 1.5 W/cm^2) for 10 min. During laser irradiation, the temperature was recorded by an infrared camera (FLIR, E6).

Preparation of MeO-TSI@F127 NPs. The powder of F127 (10 mg) was dissolved in water (1 mL) to obtain a homogenous aqueous solution, followed by the addition of MeO-TSI in THF (1 mg/mL) under stirring. The suspension was then bubbled with dry argon gas for at least 20 min to remove THF. Next, the suspension was dialyzed against deionized water with a molecular weight cut-off (MWCO) of 5000 Da for 24 h, which was then freeze-dried to give MeO-TSI@F127 nanoparticles (denoted as MeO-TSI@F127 NPs) for subsequent use.

Photothermal properties of MeO-TSI@F127 NPs. The suspensions of MeO-TSI@F127 NPs with varying concentrations of MeO-TSI (50, 100, 150, and 200 µM) were added into a cuvette with a bottom area of 1 cm². Next, the systems with different concentrations of MeO-TSI@F127 NPs were irradiated under an 808 nm laser at different power densities (0.3, 0.6, 0.9, 1.2, and 1.5 W/cm^2) for 10 min. During laser irradiation, the temperature was recorded by an infrared camera (FLIR, E6) in a real-time fashion.

Calculation of the photothermal conversion efficiency. The photothermal conversion efficiencies of MeO-TSI nanoaggregates and MeO-TSI@F127 NPs were calculated according to a previously reported method.[2] Briefly, the involved calculation equations were described as follows:

$$\eta = \frac{hS\,(\mathrm{T_{max}}\text{-}\mathrm{T_{surrounding}})\text{-}\mathrm{Q_{dis}}}{I\,(1\text{-}10^{\text{-}\mathrm{A_{808nm}}})} = \frac{hS\,(\Delta\mathrm{T_{max,surrounding}}\text{-}\Delta\mathrm{T_{H_2O,surrounding}})}{I\,(1\text{-}10^{\text{-}\mathrm{A_{808nm}}})} \quad (i)$$

$$\mathrm{Q_{dis}} = hS\Delta\mathrm{T_{H_2O,surrounding}} \quad (ii)$$

$$\theta = \frac{\mathrm{T}\text{-}\mathrm{T_{surrounding}}}{\mathrm{T_{max}}\text{-}\mathrm{T_{surrounding}}} \quad (iii)$$

$$t = \text{-}\tau\ln\theta \quad (iv)$$

$$hS = \frac{mC}{\tau} \quad (v)$$

where *h* is the heat transfer coefficient, *S* is the surface area of the container, and the value of *hS* is obtained from Equation (*v*). Q_{dis} represents the dissipated heat of the solvent. As for an aqueous system, the input heat typically equals to the output heat at the maximum steady-state temperature. As such, Equation (*ii*) could be derived, where $\Delta T_{H_2O,surrounding}$ corresponds to the temperature variation of water at the maximum steady-state temperature. According to the cooling curve after photothermal heating as shown in Figure S10A and S12A, the $\Delta T_{max,surrounding}$ of MeO-TSI nanoaggregates and MeO-TSI@F127 NPs was 14.1 °C and 15.1 °C, respectively. The $\Delta T_{H_2O,surrounding}$ of MeO-TSI nanoaggregates and MeO-TSI@F127 NPs were 0.3 °C and 0.6 °C, respectively. The laser power (*I*) was 0.3 W, while the absorbance of MeO-TSI nanoaggregates and MeO-TSI@F127 NPs at 808 nm (A_{808nm}) was 0.460 and 0.538, respectively. To gain *hS*, a dimensionless parameter θ is defined as in Equation (*iii*). Furthermore, the time constant τ for MeO-TSI nanoaggregates and MeO-TSI@F127 NPs was deduced from Equation (*iv*) (Figure S10B and S12B), which was 222.879 s and 194.084 s, respectively. According to Equation (*v*), since *m* (mass) and *C* (specific heat capacity) were

0.3 g and 4.2 J/g·°C, *hS* was calculated to be 0.0057 W/°C and 0.0065 W/°C for MeO-TSI nanoaggregates and MeO-TSI@F127 NPs, respectively. Based on the aforementioned data and Equation (*i*), the photothermal conversion efficiencies (η) of MeO-TSI nanoaggregates and MeO-TSI@F127 NPs under 808 nm laser irradiation were determined to be 40.3% and 44.2%, respectively.

Photothermal stability of MeO-TSI@F127 NPs. The suspension of MeO-TSI@F127 NPs (MeO-TSI, 100 µM) and the aqueous solution of ICG (100 µM) were subjected to repeated on-off irradiation cycles by an 808 nm laser at a power density of 1.2 W/cm^2. During laser irradiation, the temperature was recorded by an infrared camera (FLIR, E6) in a real-time fashion.

Preparation of P(NIPAM-AM) hydrogels. NIPAM (3.98 mmol, 450 mg), AM (weight ratios of NIPAM/AM = 10:1, 30:1, and 50:1), and MBA (0.06 mmol, 9 mg) were dissolved in water (5 mL) and stirred to give a homogenous solution. Next, TEMED (5µL) and KPS (0.02 mmol, 5 mg) were added under continuous stirring for uniform mixing. The resulting solution was then poured into a mold, followed by staying still at room temperature for 24 h for complete polymerization and gel formation.

Characterization of P(NIPAM-AM) hydrogels. The transmittance of P(NIPAM-AM) hydrogels at 808 nm for different temperatures was measured by a UV–Vis–NIR spectrophotometer (Shimadzu, UV-2600). Specifically, P(NIPAM-AM) hydrogels were heated from 25 to 55 °C at a heating rate of 2 °C per 10 min, in which the transmittance values were measured at a temperature interval of 2 °C.

Thermoresponsive hydrogel-enabled thermostatic control on the photothermal property of MeO-TSI nanoaggregates. The suspensions of MeO-TSI nanoaggregates were added into a cuvette with a bottom area of 1 cm^2. After the cuvette was filled up with the suspension, a coverslip was gently placed on the liquid surface, followed by putting down a P(NIPAM-AM) hydrogel on the top of the coverslip. The system was then irradiated under an 808 nm laser at a direction perpendicular to the coverslip for 10 min. For the P(NIPAM-AM) hydrogel with the weight ratio of NIPAM/AM = 10:1, five experimental groups were included, that is, *i*) MeO-TSI nanoaggregates (100 µM) + laser (0.6 W/cm^2), *ii*) MeO-TSI nanoaggregates (100 µM) + laser (0.9 W/cm^2), *iii*) MeO-TSI nanoaggregates (100 µM) + laser (1.2 W/cm^2), *iv*)

MeO-TSI nanoaggregates (150 μM) + laser (0.9 W/cm^2), and *v*) MeO-TSI nanoaggregates (150 μM) + laser (1.2 W/cm^2). For the P(NIPAM-AM) hydrogel with the weight ratio of NIPAM/AM = 30:1, one experimental group was included, that is, MeO-TSI nanoaggregates (100 μM) + laser (0.9 W/cm^2). For the P(NIPAM-AM) hydrogel with the weight ratio of NIPAM/AM = 50:1, one experimental group was included, that is, MeO-TSI nanoaggregates (100 μM) + laser (0.6 W/cm^2). During laser irradiation, the temperature was recorded by an infrared camera (FLIR, E6) in a real-time fashion.

Thermoresponsive hydrogel-enabled thermostatic control on the photothermal property of MeO-TSI@F127 NPs. The suspensions of MeO-TSI@F127 NPs were added into a cuvette with a bottom area of 1 cm^2. After the cuvette was filled up with the suspension, a coverslip was gently placed on the liquid surface, followed by putting down a P(NIPAM-AM) hydrogel on the top of the coverslip. The system was then irradiated under an 808 nm laser at a direction perpendicular to the coverslip for 10 min. For the P(NIPAM-AM) hydrogel with the weight ratio of NIPAM/AM = 10:1, one experimental group was included, that is, MeO-TSI@F127 NPs (100 μM) + laser (1.2 W/cm^2). For the P(NIPAM-AM) hydrogel with the weight ratio of NIPAM/AM = 30:1, one experimental group was included, that is, MeO-TSI@F127 NPs (100 μM) + laser (0.9 W/cm^2). For the P(NIPAM-AM) hydrogel with the weight ratio of NIPAM/AM = 50:1, one experimental group was included, that is, MeO-TSI@F127 NPs (100 μM) + laser (0.6 W/cm^2). During laser irradiation, the temperature was recorded by an infrared camera (FLIR, E6) in a real-time fashion.

Bacterial culture. The bacterial strains used in this study was methicillin-resistant *Staphylococcus aureus* (MRSA), which was provided by Prof. Jianfeng Liu (Institute of Radiation Medicine). For bacterial culture, a single colony of MRSA on a solid TSB agar plate was transferred to TSB broth medium (5 mL) with 10 μg/mL tetracycline hydrochloride in a shaking incubator (170 rpm) at 37 °C overnight. The bacterial cells were harvested by centrifugation (3500 *g*, 5 min) and washed with PBS three times. The supernatant was discarded and the remaining MRSA was resuspended in PBS, which was diluted to an optical density of 1.0 at 600 nm (OD_{600} = 1.0).

In vitro antibacterial experiments. To explore the photothermal antibacterial effect of MeO-TSI@F127 NPs *in vitro*, eight experimental groups were included, that is, *i*) the group treated with PBS, *ii*) the group treated with MeO-TSI@F127 NPs (50 μM), *iii*) the group treated with

MeO-TSI@F127 NPs (100 μM), *iv*) the group treated with MeO-TSI@F127 NPs (200 μM), *v*) the group treated with PBS + 808 nm laser (0.6 W/cm^2, 10 min), *vi*) the group treated with MeO-TSI@F127 NPs (50 μM) + 808 nm laser (0.6 W/cm^2, 10 min), *vii*) the group treated with MeO-TSI@F127 NPs (100 μM) + 808 nm laser (0.6 W/cm^2, 10 min), and *viii*) the group treated with MeO-TSI@F127 NPs (200 μM) + 808 nm laser (0.6 W/cm^2, 10 min). Next, the treated bacterial suspensions were diluted by 5 × 10^4 fold with PBS. The diluted suspensions (100 μL) were then spread on TSB plates with 10 μg/mL tetracycline hydrochloride, followed by incubation at 37 °C for 15–18 h. The colony-forming unit (CFU) number of each plate was counted, which was used for quantitative antibacterial evaluation (n = 3).

Live/dead staining assay. Live/Dead®Baclight™ bacterial viability kit was used to indicate bacterial viability, in which the bacteria with intact cell membranes will stain green, whereas the bacteria with damaged cell membranes will stain red. Once the incubation was completed as described in "*In vitro antibacterial experiments*", the bacteria were collected by centrifugation at 3500 *g* for 3 min and washed with PBS. The bacteria were then stained with SYTO 9 and propidium iodide (PI) for 15 min in the dark at final concentrations of 3.34 μM and 30 μM, respectively. The bacterial suspension (10 μL) was added to a glass slide, which was carefully covered by a clean coverslip for characterization by a confocal laser scanning microscope (Nikon, A1$^+$, SYTO 9, λex = 488 nm, λem = 490–540 nm; PI, λex = 488 nm, λem = 630–680 nm).

Morphological characterization of bacteria. Once the incubation was completed as described in "*In vitro antibacterial experiments*", the bacteria were collected by centrifugation at 3500 *g* for 3 min and washed with PBS. The bacteria were fixed with 2.5% glutaraldehyde at room temperature for 30 min. The bacteria were then collected by centrifugation at 3500 *g* for 10 min, washed with PBS, and resuspended in PBS. The bacterial suspensions (5 μL) were added to clean silicon wafers followed by air drying. Immediately the samples became dried, the silicon wafers were immersed in 2.5% glutaraldehyde and fixed at room temperature for 3 h. After washing with water two times, the bacterial cells were subjected to gradient ethanol dehydration from 30% to 100%, including 30%, 50%, 70%, 80%, 90%, and 100% (2 × 6 min for each concentration). After the samples were fully dried, they were coated with gold before examination by a scanning electron microscope (SEM, ZEISS, MERLIN Compact).

Cell culture. NIH-3T3 cells, a mouse fibroblast cell line, were cultured in high-glucose DMEM containing 10% fetal bovine serum (FBS) and 1% antibiotics (100 units/mL penicillin and 100 g/mL streptomycin) in a 5% CO_2 humidity incubator at 37 °C. The culture medium was changed every other day.

Cell viability assay. The viability of NIH-3T3 cells incubated with different concentrations of MeO-TSI@F127 NPs was evaluated by the MTT assay. Specifically, NIH-3T3 cells were seeded in a 96-well plate at a density of 8000–10000 cells per well and cultured overnight. Next, MeO-TSI@F127 NPs with varying concentrations of MeO-TSI (50, 100, 150, 200, and 250 μM) were added into the cells, followed by incubation at 37 °C for 12 h. The cells were replenished with fresh culture medium and incubated for another 4, 10, and 24 h, respectively. Subsequently, the MTT solution (0.5 mg/mL in medium, 100 μL per well) was added to each well and incubated at 37 °C for 4 h. After removing the MTT solution, DMSO (150 μL per well) was added to dissolve the formed formazan crystals. The absorbance values at 490 nm were recorded by a microplate reader (Tecan, Infinite M Nano). The cell viability was defined as the percentage of surviving cells versus untreated cells (n = 3).

Temperature-dependent cell viability. The viability of NIH-3T3 cells incubated with MeO-TSI@F127 NPs under different photothermal equilibrium temperatures for different periods of time was evaluated by the MTT assay. NIH-3T3 cells were first seeded in a 96-well plate at a density of 8000–10000 cells per well and cultured overnight. Next, MeO-TSI@F127 NPs (100 μM) were added into the cells, followed by exposure to an 808 nm laser (0.6 mW/cm^2) for 5 and 10 min, respectively. To further elevate the photothermal equilibrium temperature, the cells with added MeO-TSI@F127 NPs (100 μM) were exposed to an 808 nm laser (1.2 mW/cm^2) for 5 and 10 min, respectively. Subsequently, the MTT solution (0.5 mg/mL in medium, 100 μL per well) was added to each well and incubated at 37 °C for 4 h. After removing the MTT solution, DMSO (150 μL per well) was added to dissolve the formed formazan crystals. The absorbance values at 490 nm were recorded by a microplate reader. The cell viability was defined as the percentage of surviving cells versus untreated cells (n = 3).

In vivo antibacterial experiments. All animal experiments were reviewed and approved by the Institutional Animal Care and Use Committee (IACUC) of Nankai University (Permit No. 2021-SYDWLL-000001). Specifically, female BALB/c mice (6–8 weeks old) were purchased

from SPF Biotechnology Co., Ltd. (Beijing, China) and raised in the specific-pathogen-free (SPF) grade laboratory. Prior to the creation of skin injury, the mice were intraperitoneally injected with cyclophosphamide (80 mg/kg) to compromise their immunity against the inoculated bacteria. At 24 h post injection, a round wound with a diameter of 0.8 cm was created in the right back of the mouse using surgical scissors, followed by wound infection with MRSA (100 μL, OD_{600} = 0.8). The wound of each mouse was then bounded with gauze, and the day of the bacterial inoculation was defined as the zero day. At 24 h post inoculation, the infected mice were randomly divided into five groups (n = 6), and different liquids (50 μL) were added onto the wound area for different treatments, including *i*) the group treated with PBS, *ii*) the group treated with PBS + 808 nm laser (1.2 W/cm^2, 5 min), *iii*) the group treated with MeO-TSI@F127 NPs (150 μM), *iv*) the group treated with MeO-TSI@F127 NPs (150 μM) + 808 nm laser (1.2 W/cm^2, 5 min), and *v*) the group treated with MeO-TSI@F127 NPs (150 μM) + 808 nm laser (1.2 W/cm^2, 5 min) + P(NIPAM-AM) hydrogel (NIPAM/AM = 10:1). During laser irradiation, the temperature was dynamically monitored using the FLIR E6 infrared camera (FLIR Systems, Inc., Wilsonville, USA). The healing process of the infected areas and body weights of the infected mice were recorded by a digital camera and balance throughout the therapeutic window (11 days), in which the damaged areas were measured and quantified by the ImageJ software (National Institutes of Health, Bethesda, MD). To assess the *in vivo* therapeutic effect and biosafety issue, the mice were sacrificed at the end of the treatment, and half of the infected skin tissues as well as the major organs (including heart, liver, spleen, lung, and kidney) were collected and fixed in 4% paraformaldehyde for 12 h. All these tissues were subjected to hematoxylin and eosin (H&E) staining, and the infected skin tissues were subjected to immunohistochemical staining on CD31 (a marker of endothelial cell for vascular differentiation), type I collagen (COL I), and type III collagen (COL III), respectively. To determine the number of bacteria at the infection site, the other half of the infected skin tissue was homogenized in PBS (500 μL), and the diluted tissue suspensions (1 × 10^2 fold) were used for plate counting after incubation at 37 °C for 20 h (n = 3). Notably, to preliminarily evaluate the antibacterial effects at the infected areas at day 2, one mouse from each group was sacrificed, and half of the infected skin tissues were homogenized in PBS (500 μL), and the diluted tissue suspensions (1 × 10^4 fold) were used for plate counting after incubation at 37 °C for 20 h (n = 3). The other half the infected skin tissues were subjected to H&E staining.

Statistical analysis. Statistical analysis was conducted using one-way analysis of variance (ANOVA) with Tukey's test by OriginPro 8.1 (*$p < 0.05$; **$p < 0.01$; ***$p < 0.001$). The results were expressed as the mean ± standard deviation, with "*n*" indicating the number of samples per group.

References

[1] J. Liu, L. Chen, S. Shao, Z. Xie, Y. Cheng, Y. Geng, L. Wang, X. Jing, F. Wang, *J. Mater. Chem.* **2008**, *18*, 319.
[2] H. Lin, S. Gao, C. Dai, Y. Chen, J. Shi, *J. Am. Chem. Soc.* **2017**, *139*, 16235.
[3] Y. Wang, Y. Yang, Y. Shi, H. Song, C. Yu, *Adv. Mater.* **2020**, *32*, 1904106.
[4] D. S. Karaman, U. K. Ercan, E. Bakay, N. Topaloglu, J. M. Rosenholm, *Adv. Funct. Mater.* **2020**, *30*, 1908783.

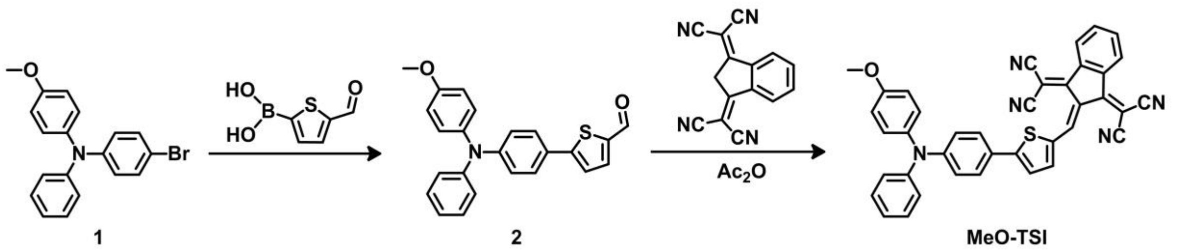

Figure S1. Synthetic route to MeO-TSI.

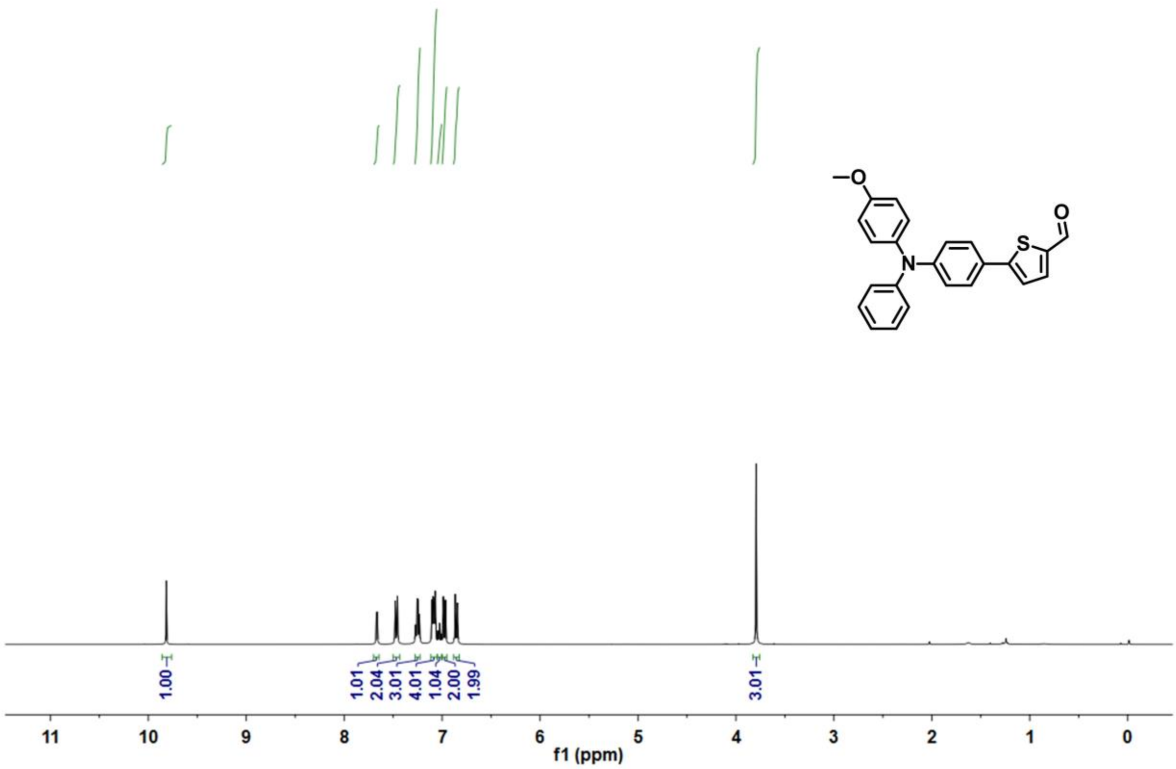

Figure S2. ^{1}H-NMR spectrum of compound **2** in $CDCl_3$.

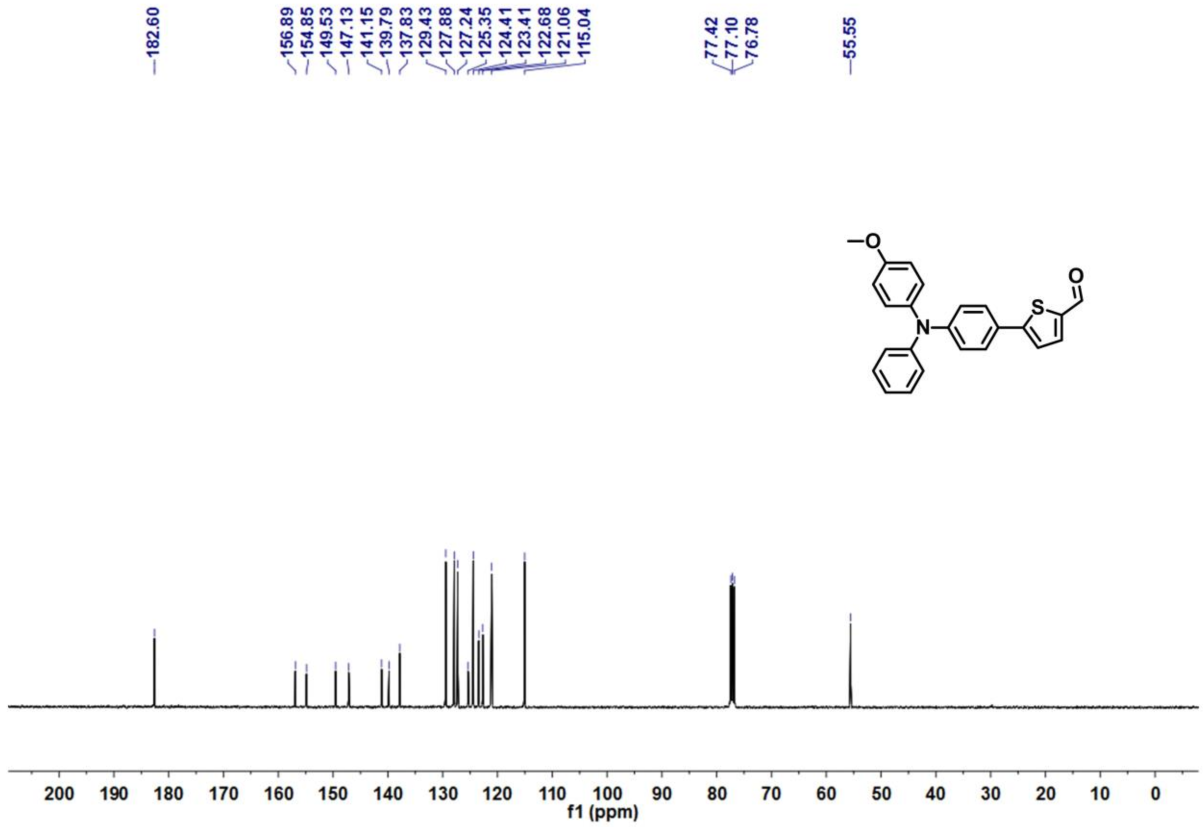

Figure S3. ^{13}C-NMR spectrum of compound **2** in $CDCl_3$.

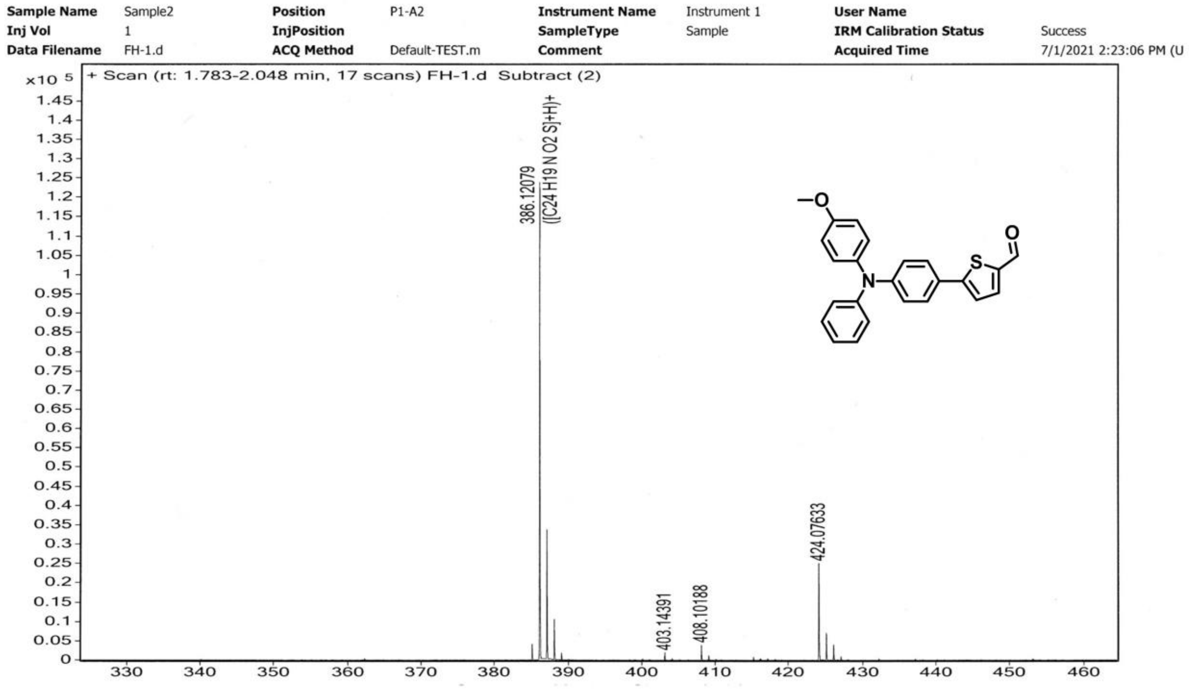

Figure S4. HRMS spectrum of compound **2**.

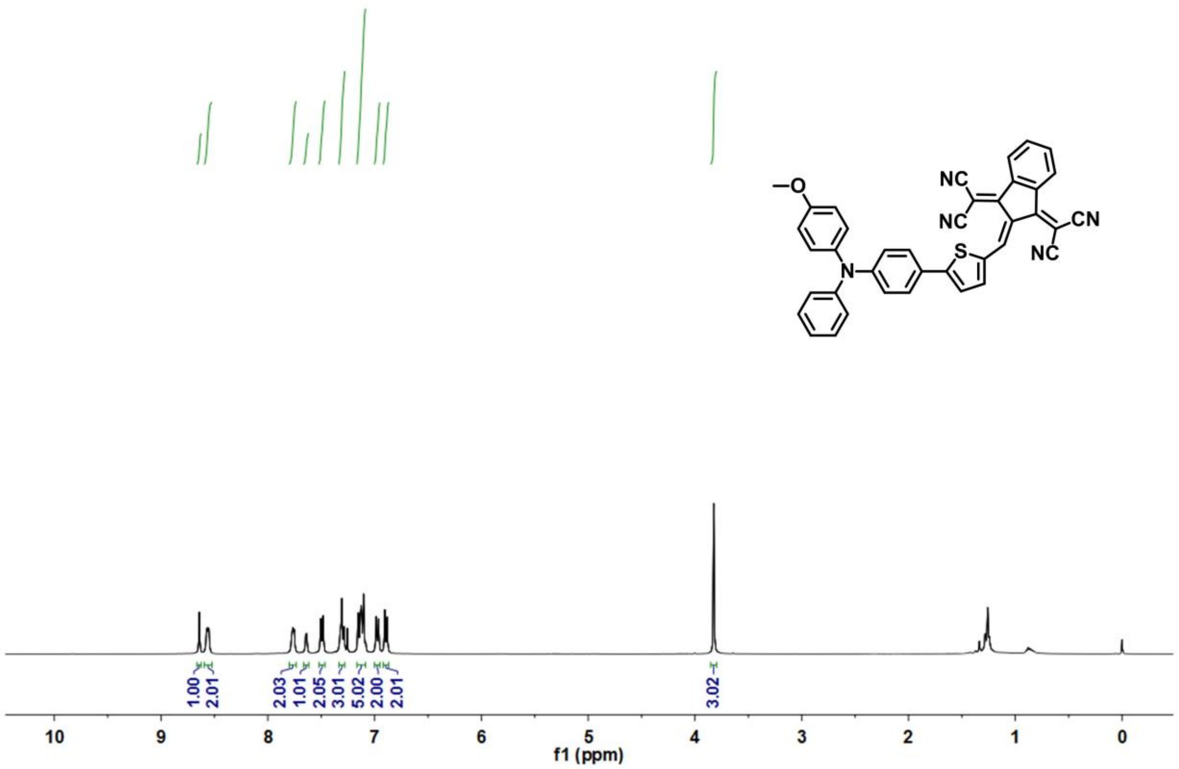

Figure S5. ^{1}H-NMR spectrum of MeO-TSI in $CDCl_3$.

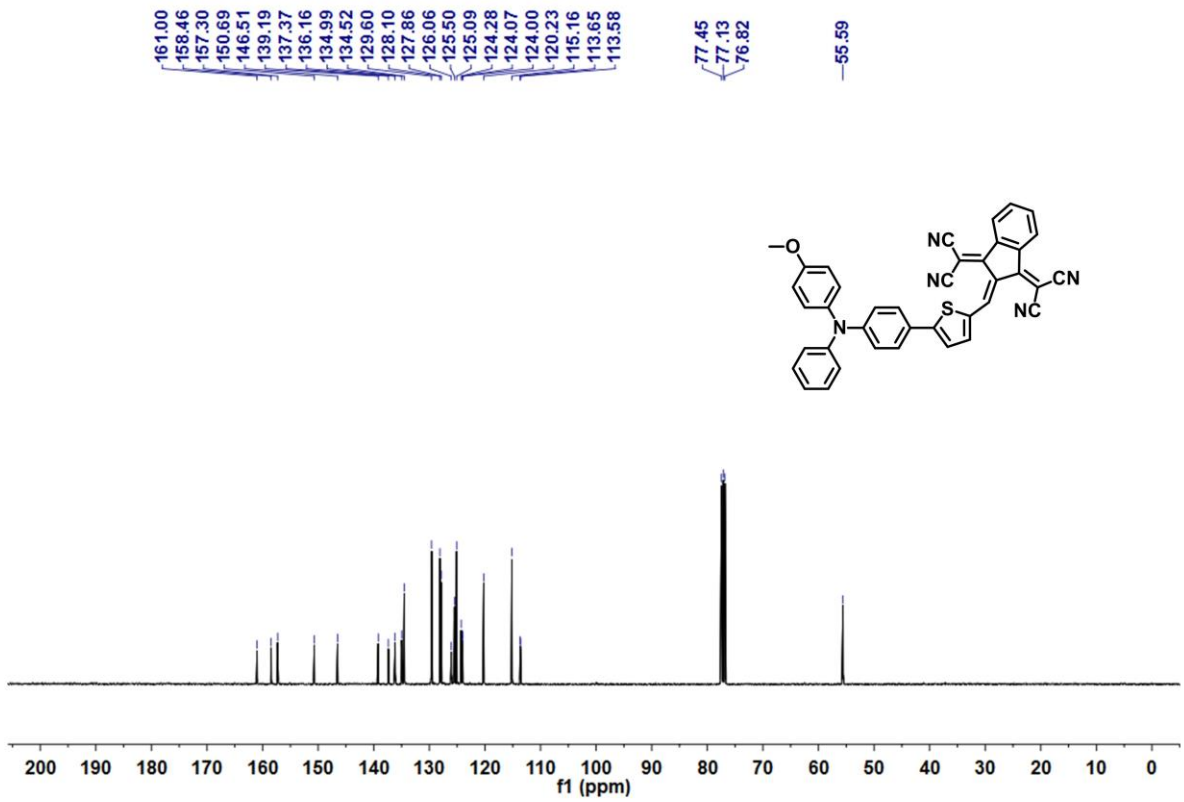

Figure S6. ^{13}C-NMR spectrum of MeO-TSI in $CDCl_3$.

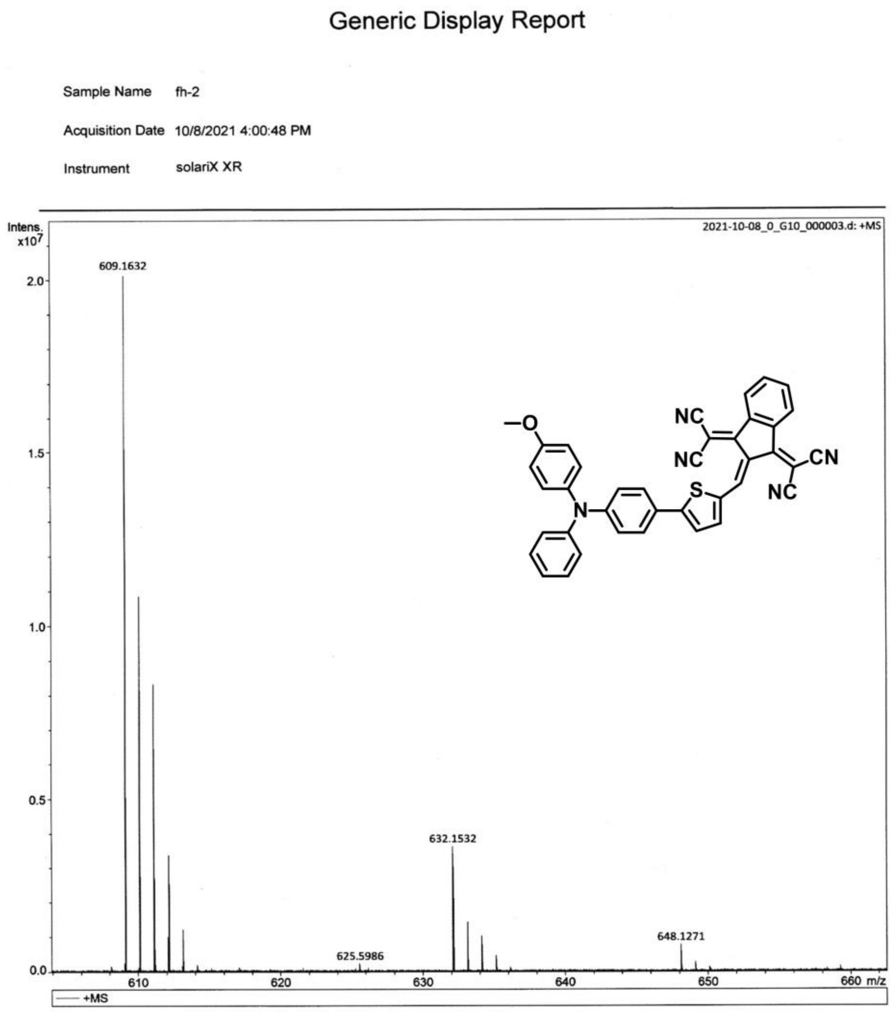

Figure S7. HRMS spectrum of MeO-TSI.

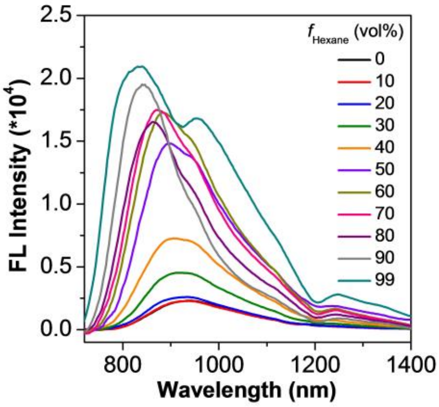

Figure S8. Emission spectrum of MeO-TSI in the mixture of $CHCl_3$/hexane. λex = 638 nm.

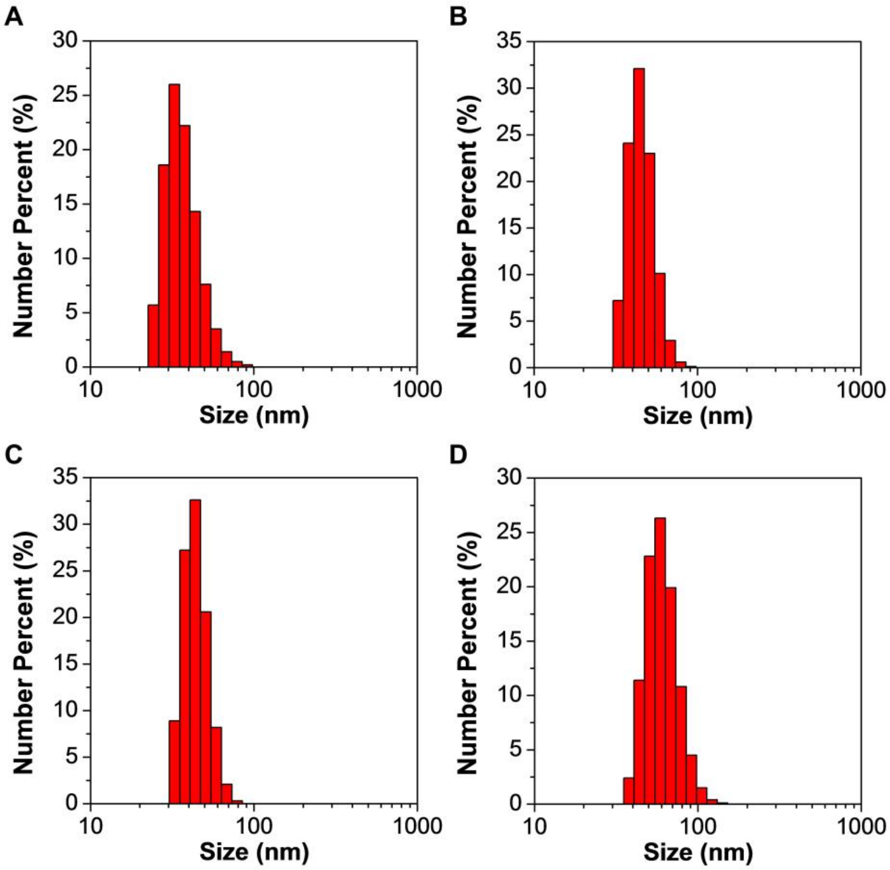

Figure S9. Size distribution of MeO-TSI nanoaggregates measured by DLS. A) 50 µM. B) 100 µM. C) 150 µM. D) 200 µM.

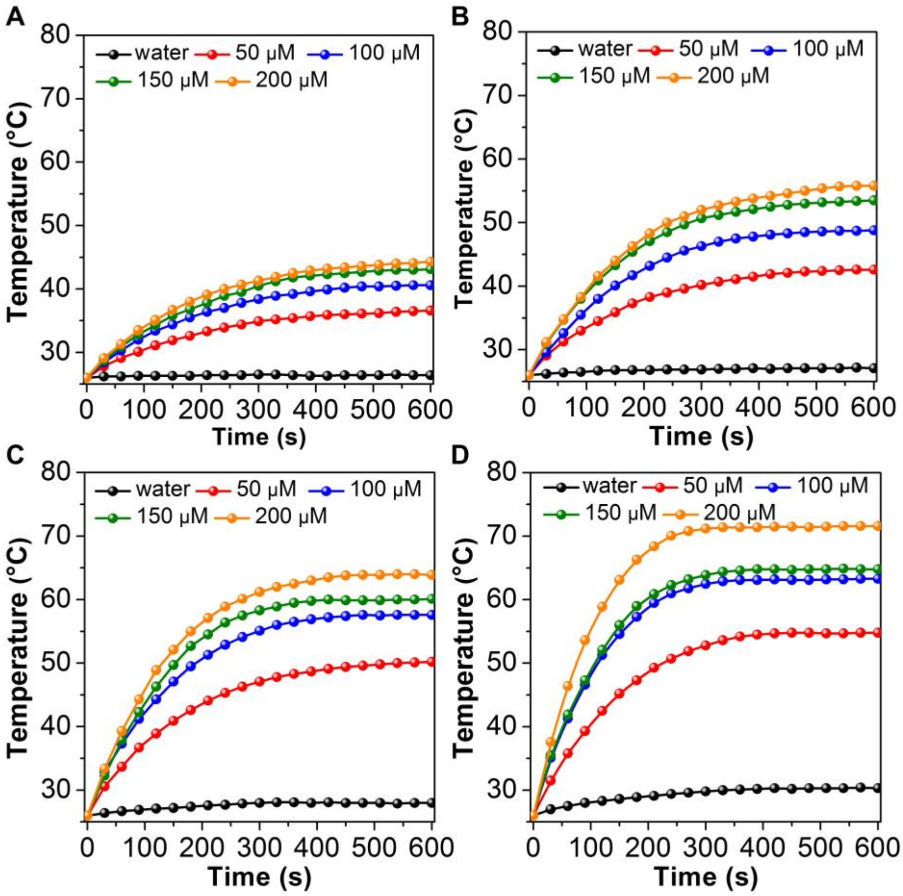

Figure S10. Photothermal curves of MeO-TSI nanoaggregates with varying concentrations of MeO-TSI (50, 100, 150, and 200 μM) in response to 808 nm laser irradiation at different power densities for 10 min. A) 0.3 W/cm^2. B) 0.6 W/cm^2. C) 0.9 W/cm^2. D) 1.5 W/cm^2.

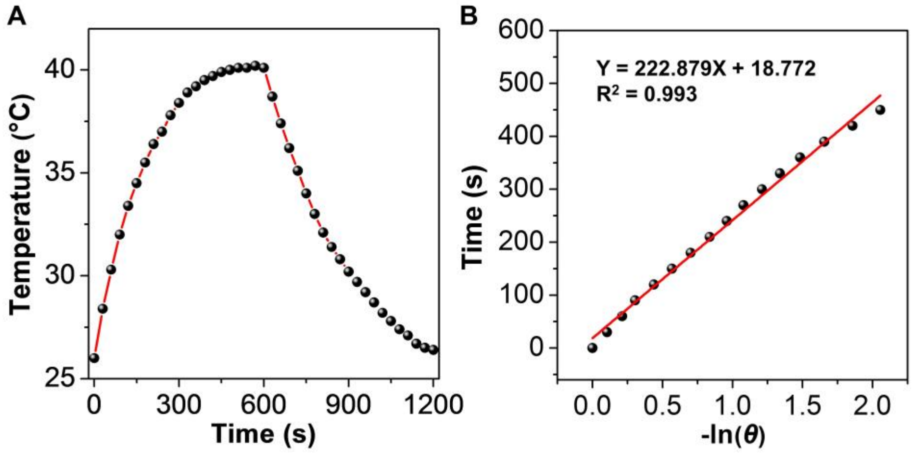

Figure S11. Calculation of the photothermal conversion efficiency of MeO-TSI nanoaggregates in water. A) Temperature variation of MeO-TSI nanoaggregates irradiated by an 808 nm laser (0.3 W/cm^2) for 10 min, followed by natural cooling. B) Linear fitting line of time as a function of negative natural logarithm of the driving force temperature (-ln θ) obtained from the cooling curve of MeO-TSI nanoparticles.

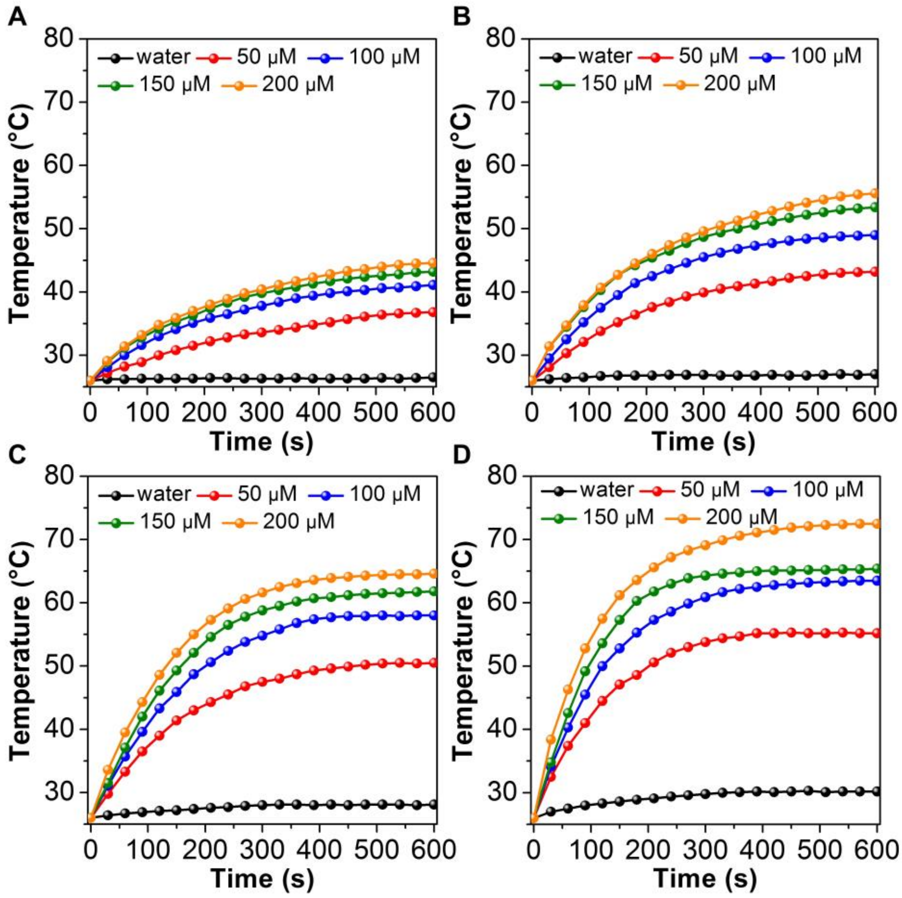

Figure S12. Photothermal curves of MeO-TSI@F127 NPs with varying concentrations of MeO-TSI (50, 100, 150, and 200 μM) in response to 808 nm laser irradiation at different power densities for 10 min. A) 0.3 W/cm^2. B) 0.6 W/cm^2. C) 0.9 W/cm^2. D) 1.5 W/cm^2.

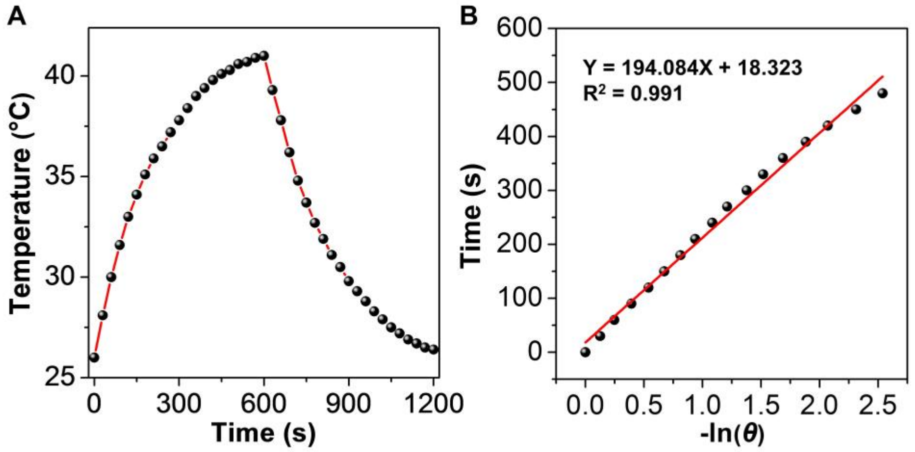

Figure S13. Calculation of the photothermal conversion efficiency (η) of MeO-TSI@F127 NPs in water. A) Temperature variation of MeO-TSI@F127 NPs irradiated by 808 nm laser (0.3 W/cm^2) for 10 min, followed by natural cooling. B) Linear fitting line of time as a function of negative natural logarithm of the driving force temperature (-ln θ) obtained from the cooling curve of MeO-TSI@F127 NPs.

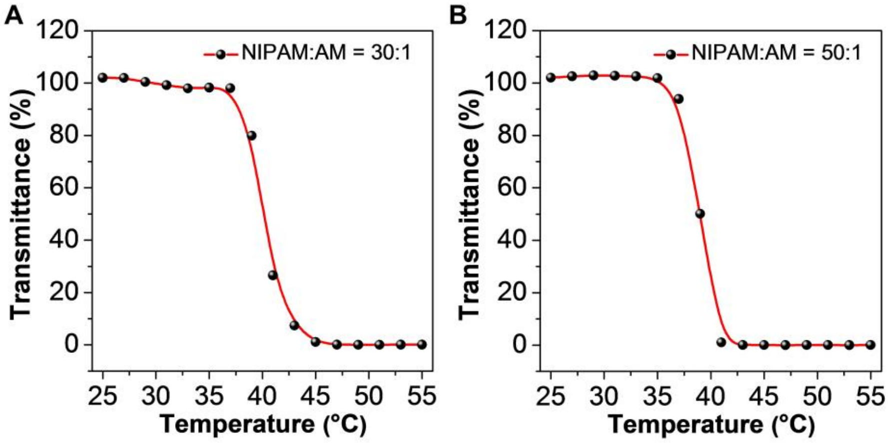

Figure S14. Transmittance of the P(NIPAM-AM) hydrogels at 808 nm as a function of temperature. A) NIPAM:AM = 30:1. B) NIPAM:AM = 50:1.

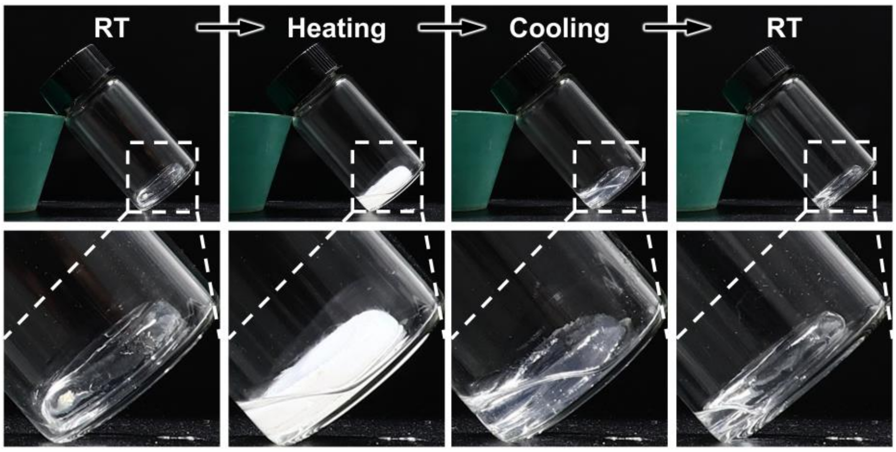

Figure S15. Photographs showing the reversible phase-change process of the P(NIPAM-AM) hydrogel (NIPAM:AM = 10:1).

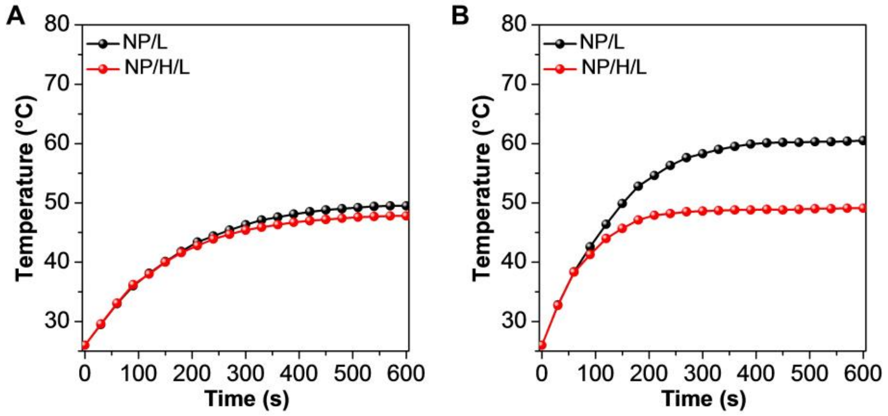

Figure S16. Photothermal curves of MeO-TSI@F127 NPs in the absence (NP/L) and presence (NP/H/L) of a P(NIPAM-AM) hydrogel (NIPAM:AM = 10:1) under 808 nm laser irradiation. A) MeO-TSI@F127 NPs (100 µM) + laser (0.6 W/cm^2). B) MeO-TSI@F127 NPs (150 µM) + laser (0.9 W/cm^2).

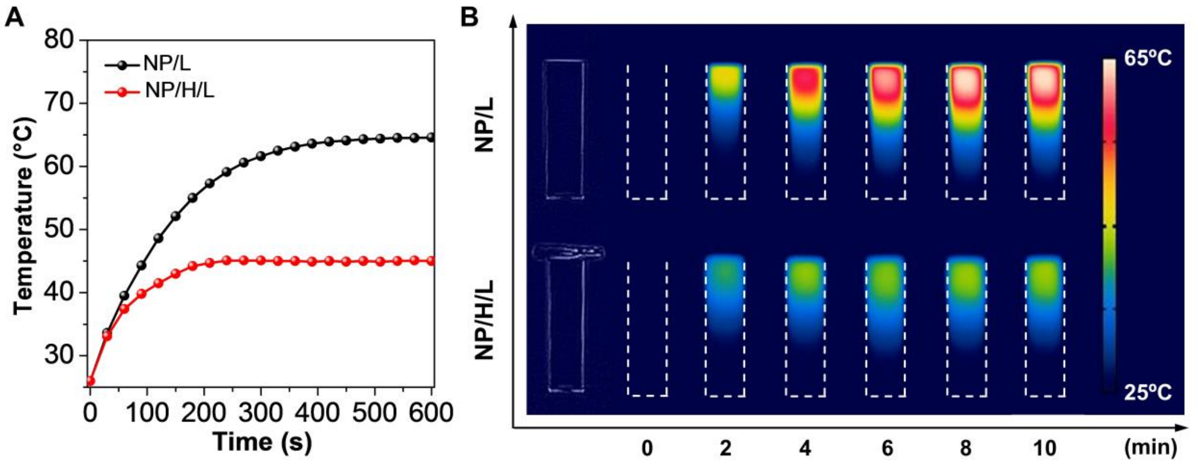

Figure S17. Photothermal properties of MeO-TSI@F127 NPs (200 μM) in the absence (NP/L) and presence (NP/H/L) of a P(NIPAM-AM) hydrogel (NIPAM/AM = 30:1) under 808 nm laser irradiation (0.9 W/cm^2). A) Photothermal curves of MeO-TSI@F127 NPs. B) Thermal images of MeO-TSI@F127 NPs.

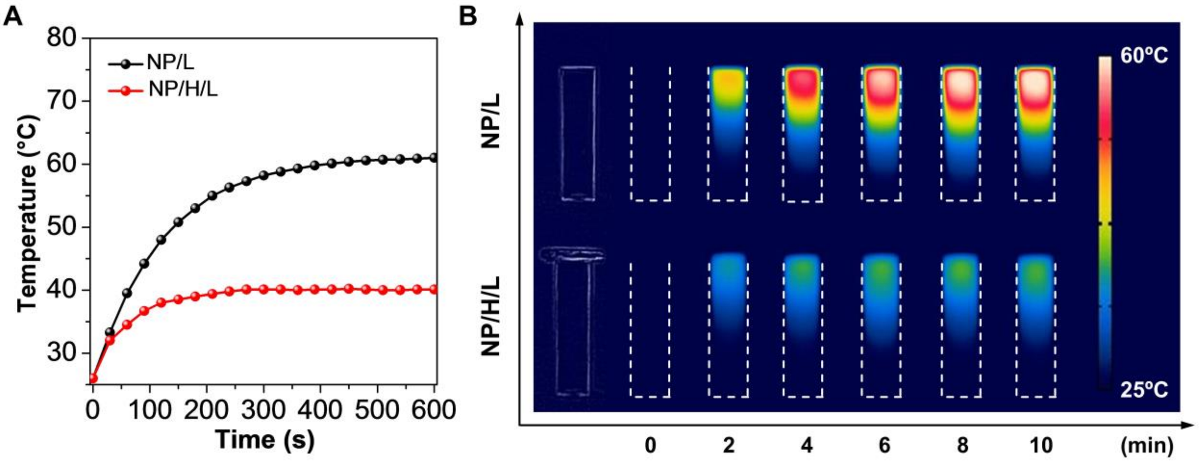

Figure S18. Photothermal properties of MeO-TSI@F127 NPs (100 µM) in the absence (NP/L) and presence (NP/H/L) of a P(NIPAM-AM) hydrogel (NIPAM/AM = 50:1) under 808 nm laser irradiation (1.2 W/cm^2). A) Photothermal curves of MeO-TSI@F127 NPs. B) Thermal images of MeO-TSI@F127 NPs.

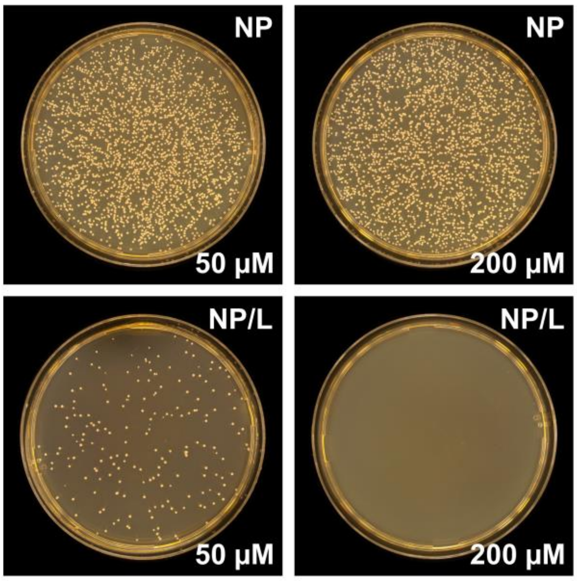

Figure S19. Photographs of *in vitro* antibacterial behaviors for different groups toward MRSA upon NIR irradiation. “NP” and “NP/L” represent the group treated with MeO-TSI@F127 NPs and the NIR-irradiated “NP” group, respectively.

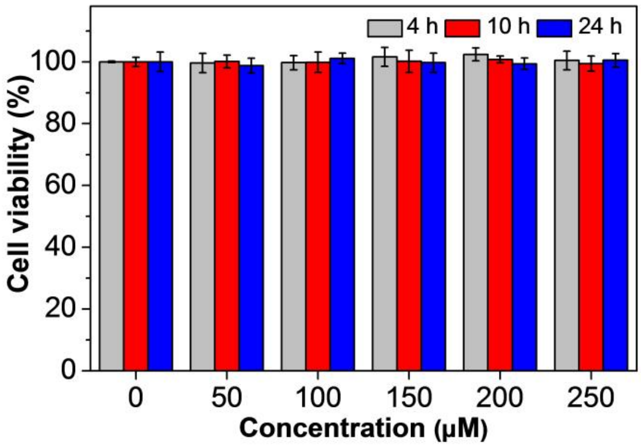

Figure S20. Viability of NIH-3T3 cells incubated with MeO-TSI@F127 NPs with varying concentrations of different concentrations for 4, 10, and 24 h, respectively (*n* = 3).

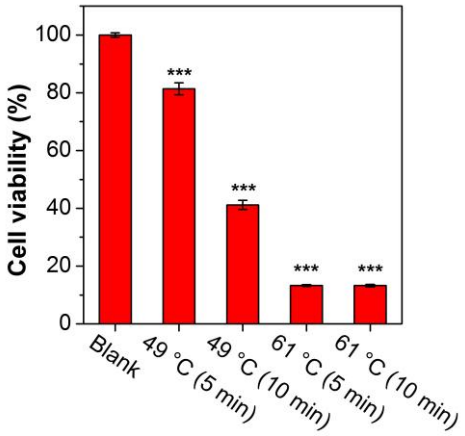

Figure S21. Viability of NIH-3T3 cells incubated with MeO-TSI@F127 NPs for different periods of time with the photothermal equilibrium temperatures of *ca.* 49 and 61 °C, respectively (n = 3). ***$p < 0.001$ when compared with the "Blank" group.

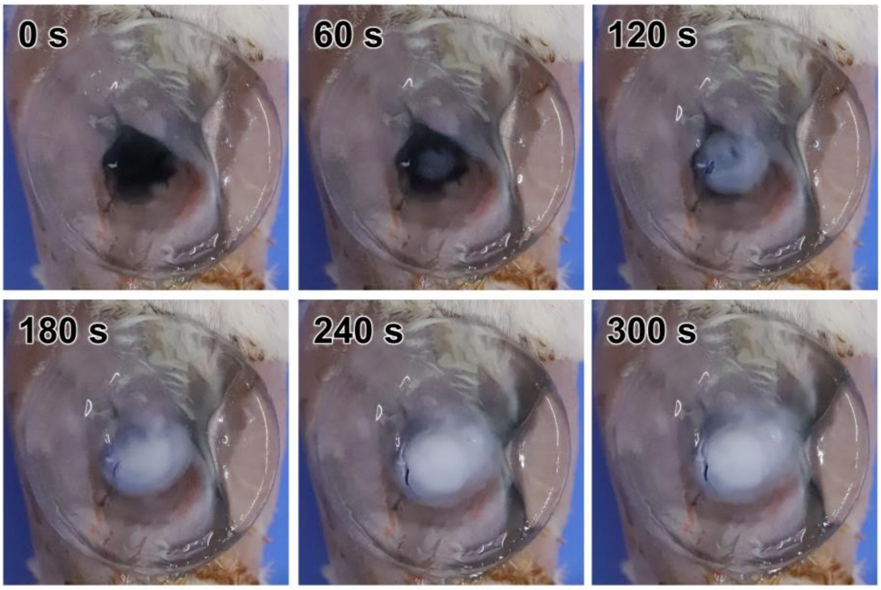

Figure S22. Photographs of the phase-transition process of P(NIPAM-AM) hydrogel (NIPAM/AM = 10:1) during 808 nm laser irradiation for thermostatic photothermal therapy of a MRSA-infected wound in a BALB/c mouse.

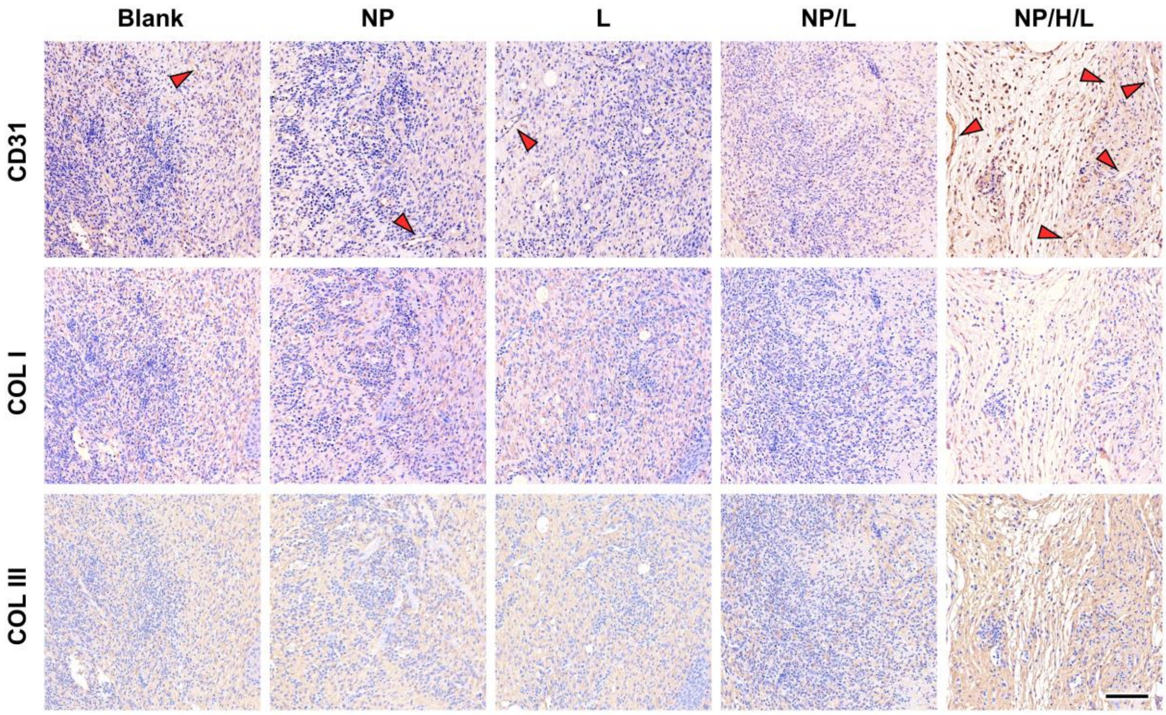

Figure S23. Immunohistochemical staining analysis of infected wounds under different treatments. Red arrowheads indicate angiogenesis. Scale bar: 100 μm.

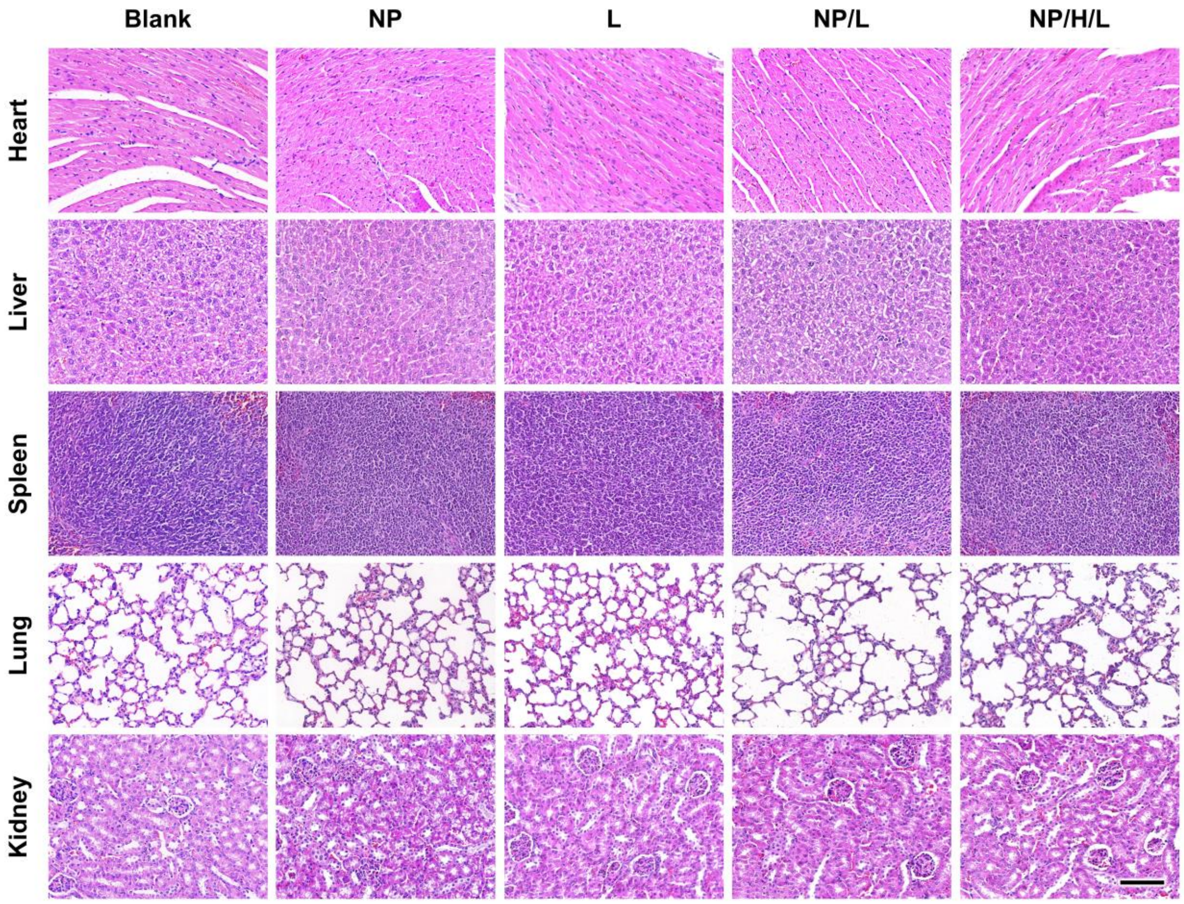

Figure S24. Histological analysis of different organs (heart, liver, spleen, lung, and kidney) from MRSA-infected mice stained by H&E after the completion of antibacterial experiments. Scale bar: 100 μm.

Table S1. Common antibiotic-independent antibacterial strategies[3,4]

Strategies	Advantages	Disadvantages
Surgery	Precise and rapid removal of infected tissues	Tissue/organ damage<br>Pain and blood loss
Materials with intrinsic antibacterial activity	Multiple antibacterial mechanisms<br>Broad-spectrum antibacterial effect<br>Easy surface modification for nanomaterials	Toxicity to mammalian cells (*e.g.*, metal-based materials, antimicrobial peptides, and cationic polymers)
Photodynamic therapy	Non-invasiveness<br>High spatiotemporal resolution<br>Broad-spectrum antibacterial effect<br>Low probability to induce drug resistance	Poor efficacy in hypoxic environments
Photothermal therapy	Non-invasiveness<br>High spatiotemporal resolution<br>Broad-spectrum antibacterial effect<br>Easy eradication of drug-resistant bacteria	Thermal damages to healthy tissues/organs
